# Close or Distant Past? The Role of Temporal Distance in Responses to Intergroup Violence From Victim and Perpetrator Perspectives

**DOI:** 10.1177/0146167220945890

**Published:** 2020-08-01

**Authors:** Mengyao Li, Bernhard Leidner, Nebojša Petrović, Nedim Prelic

**Affiliations:** 1 University of Massachusetts Amherst, USA; 2 Max Planck Institute for Research on Collective Goods, Germany; 3 University of Belgrade, Serbia; 4 University of Tuzla, Bosnia and Herzegovina

**Keywords:** intergroup violence, temporal distance, justice, reconciliation, ingroup glorification

## Abstract

In two different intergroup contexts, three studies investigated the role of temporal distance in responses to intergroup violence from both victim and perpetrator perspectives. In the context of the conflict between Serbs and Bosniaks, Study 1 showed that whereas increased subjective temporal distance predicted less support for justice-restoring efforts and less outgroup empathy among the perpetrator group (Serbs), it predicted more conciliatory, pro-outgroup attitudes among the victim group (Bosniaks). Furthermore, Bosniaks perceived the war as temporally closer than did Serbs. In the context of the U.S.–Iran conflict, Study 2 provided a partial conceptual replication of Study 1 and demonstrated that ingroup glorification motivated more temporal distancing among perpetrators and less temporal distancing among victims. Study 3 further established the causal effects of temporal distance on intergroup outcomes, and that these effects were moderated by glorification. Implications for post-conflict peacebuilding are discussed.

Global politics in the post World War II period has been characterized by an uptake in the establishment of international norms, laws, and institutions to address past and prevent future human rights violations. Despite the marked advancement in the international justice system, a persistent challenge facing societies recovering from mass atrocities concerns the timing of pursuing justice (e.g., Fletcher et al., 2009). In the case of retributive justice, for example, putting perpetrators on trial immediately after the conflict might threaten a recent and fragile transition from violence to peace (e.g., Zalaquett, 1990). On the contrary, failure to provide an effective and timely response to victims’ strong need for justice can hinder forgiveness and obstruct reconciliation efforts in the long run (e.g., Backer, 2010; Li et al., 2018; Wenzel et al., 2018). The question at stake, therefore, is what role the passage of time plays in victims’ and perpetrators’ responses to large-scale intergroup violence.

A related and equally important question concerns *subjective perceptions of time*. While related to the actual passage of time, people’s experience of temporal distance can be influenced by factors other than calendar time (Ross & Wilson, 2002). Thus, group members’ subjective perceptions of temporal distance might diverge depending on the specific role of the group in the conflict (i.e., victim or perpetrator). To our knowledge, no psychological research to date has systematically explored the role of temporal distance (be it subjective or objective) in intergroup violence from the perspectives of both victim and perpetrator groups. In an effort to address this gap in the literature, the current research aimed to examine (a) whether temporal distance from a past conflict differentially predicts victim and perpetrator group members’ attitudes toward justice and reconciliation, as well as empathy toward the outgroup; (b) whether victim and perpetrator group members also differ in their *subjective* perceptions of temporal distance; and (c) the moderating role of ingroup glorification.

## Temporal Distance and Responses to Intergroup Violence

### Perpetrator Group

When faced with wrongdoings committed by the ingroup, people tend to disengage from the immorality (e.g., Bandura, 1999; Leidner et al., 2010) or even actively moralize the wrongdoing (e.g., Leidner & Castano, 2012) as an effort to defend the ingroup. In the current research, we argue that temporal distance serves an ingroup-defensive or even morally disengaging function for members of the perpetrator group. As time (objectively or subjectively) distances people from their ingroup’s wrongdoings, they may be less interested in efforts aiming at addressing the past atrocities and mending the relationship with the victim group.

Consistent with this hypothesis, public opinion polls in Serbia suggest that the Serbian attitude toward war crimes committed during the Balkan wars and the International Criminal Tribunal for the Former Yugoslavia (ICTY) is characterized by growing confusion and decreased interest over time (Dimitrijevic, 2008). Similarly, when the Holocaust was *perceived* as more remote in time, Germans experienced less collective guilt, which in turn predicted less willingness to make amends (Peetz et al., 2010). Perpetrator group members also tend to increase their expectations of forgiveness from victims when a transgression is perceived as temporally distant rather than close (Greenaway et al., 2012). These findings collectively suggest that increased temporal distance can reduce the psychological impact of past transgressions on perpetrator group members, thus leading to less support for justice and reconciliation efforts. By the same token, increased temporal distance from past wrongdoings might also reduce perpetrator group members’ general concern for the victim group, for example, manifesting as lowered outgroup empathy. Substantial research has demonstrated the close link between empathy and attitudes toward justice and reconciliation (e.g., Greenaway et al., 2012; Noor et al., 2008). Thus, we also examined the implications of temporal perceptions for intergroup empathy.

### Victim Group

People often hear the advice that time will eventually heal the wound. Research on interpersonal conflict lends support to this popular lay theory—both objective and subjective temporal distance from an interpersonal transgression increases the victim’s willingness to forgive the perpetrator (Wohl & McGrath, 2007). Extending this finding to the intergroup context, it seems plausible that temporal distance also serves a “healing” function for victim group members. As time distances people from the ingroup’s past suffering, they may be more willing to let go of the injustices and move on. Temporal distance, therefore, can potentially influence the victim group in a similar way as it influences the perpetrator group—in the sense that it might serve to reduce the psychological impact of the group’s experiences in the conflict. However, the societal implications of such temporal distancing among the victim group should be entirely different from those among the perpetrator group. Whereas a “time-induced” reduction in the psychological impact of the ingroup’s conflict experiences should lead to more negative intergroup attitudes and less support for justice and reconciliation among the perpetrator group, it should lead to more conciliatory intergroup attitudes among the victim group. As time separates the victim group from their past suffering, they may even be willing to forgo punitive forms of justice to maintain peaceful coexistence with the perpetrator group. In addition, more temporal distance from the past might also help restore victims’ positive feelings toward the outgroup, therefore increasing their empathy toward perpetrator group members.

We have so far argued that (objective and subjective) temporal distance can have different implications for group members’ support for justice and reconciliation and general intergroup attitudes (i.e., empathy), depending on whether the ingroup has primarily committed or suffered violence in the conflict. Not only does temporal distance affect how people perceive past events, people are also able to regulate perceptions of temporal distance in self-defensive ways (Peetz et al., 2010; Ross & Wilson, 2002). Victim and perpetrator group members might therefore have asymmetric subjective perceptions of time.

## Motivated Subjective Perceptions of Temporal Distance

Past research has demonstrated that individuals *feel* farther from past experiences with negative implications for the current self-image than experiences with flattering implications (Ross & Wilson, 2002). At the group level, threat elicited by ingroup-perpetrated atrocities has been shown to increase temporal distancing from the atrocities, compared with when the threat was mitigated (Peetz et al., 2010). Although no research to date has examined perception of time as a motivated process among victim group members, tangential evidence suggests that victims might perceive past suffering as relatively close in time to actively pursue justice. In South Africa, for example, longitudinal data revealed that victims’ approval of conditional amnesty dropped dramatically in 2008 relative to 2002–2003, and the sharp decline was accompanied by an increased desire for criminal accountability even at the risk of political instability (Backer, 2010). Similarly, Bosniaks in general held rather positive views of the ICTY and domestic trials, presenting a stark contrast to the prevailing negative opinion of (Christian) Serbs (Biro et al., 2004).

The differences in victim and perpetrator groups’ responses to past conflict can potentially stem from the discrepancy in their subjective perceptions of temporal distance from the conflict. Although objective calendar time and subjective experiences of time are often closely related, what matters most in intergroup conflicts is arguably the motivated subjective perception of time. It is plausible that whereas perpetrators are motivated to temporally distance themselves from past atrocities, victims are motivated to perceive the same violent events as closer in time and thus need significantly more time to move on from the past. If perpetrator and victim groups’ divergent perceptions of temporal distance are motivated by their respective need to defend the ingroup, it stands to reason that the extent to which people identify with their own group should moderate these ingroup-defensive tendencies.

## The Moderating Role of Ingroup Glorification

Recent research on social identification has distinguished between ingroup attachment and glorification (Roccas et al., 2006). Whereas attachment refers to one’s perceived importance of and commitment to the ingroup, glorification refers to beliefs in the superiority of the ingroup and emphasizes unconditional deference to ingroup norms and authorities. Research has revealed that glorification, but not attachment, is associated with a variety of ingroup-defensive reactions to intergroup conflict, such as denial of collective guilt and ingroup responsibility for ingroup wrongdoings (Bilali, 2013; Roccas et al., 2006), heightened demands for retributive justice among victim group members (Li et al., 2018), as well as support for future violence (Li et al., 2016). In contrast, individuals who do not glorify their group tend to hold rather critical attitudes toward ingroup-committed harm and are open to restoring the relationship with the outgroup (e.g., Bilali, 2013; Leidner & Castano, 2012; Li et al., 2018).

**Figure 1.**
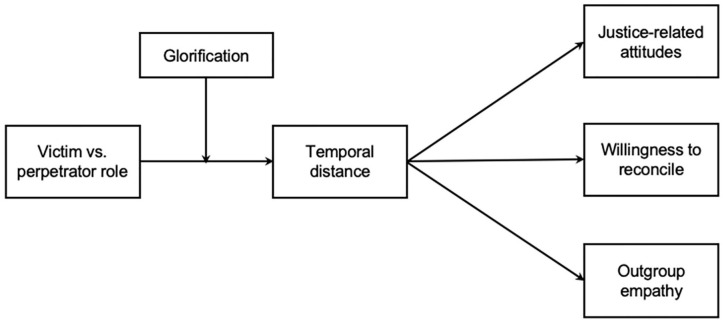
Overall conceptual model depicting the relationships between temporal distance and intergroup outcomes, as jointly affected by ingroup role as victim or perpetrator and ingroup glorification.

In line with the past research on glorification, we propose that people are motivated to temporally distance from, or remain close to, past intergroup violence to the extent that they glorify their own group. Individuals who strongly glorify their ingroup should be motivated to perceive ingroup-committed violence as temporally distant and ingroup-suffered violence as temporally close. Low glorifiers, by contrast, should be unlikely to exhibit the same ingroup-defensive pattern in their subjective perceptions of time. If anything, they might even perceive ingroup-committed violence as temporally closer in an effort to address the violence, and ingroup-suffered violence as more temporally distant to restore the relationship with the outgroup. Not only should glorification motivate subjective perceptions of temporal distance, it should also modulate the influence of time on intergroup attitudes. Given the ingroup-defensive nature of glorification, strongly (but not weakly) glorifying perpetrator group members might be particularly prone to using temporal distancing as a moral disengagement strategy, and thus become less interested in justice and reconciliation as time separates them from the ingroup’s past transgression. On the other contrary, the conciliatory or healing effects of temporal distance on victim group members should be particularly strong among low (but not high) glorifiers. Figure 1 displays the overall conceptual model, in which ingroup role as victim or perpetrator and glorification jointly influence subjective perceptions of temporal distance, and the downstream effects of temporal distance on attitudes toward justice and reconciliation, as well as outgroup empathy.

## Overview of the Present Research

In sum, we propose that victim and perpetrator group members diverge in their temporal perspectives in two ways. First, temporal distance from a past transgression will differentially predict support for justice and reconciliation, as well as empathy toward the outgroup. Second, victim and perpetrator group members will be motivated to have differential subjective perceptions of temporal distance. Furthermore, both types of temporal asymmetry will be moderated by ingroup glorification. We tested these hypotheses across two different intergroup contexts. Against the backdrop of the conflict between Serbs and Bosniaks, Study 1 examined how ingroup role influenced subjective perceptions of temporal distance and their downstream relationships with support for justice, willingness to reconcile, and empathy. In the context of the Iran–U.S. conflict, Study 2 aimed to provide a conceptual replication of Study 1, and to further test whether ingroup glorification moderated the effects of ingroup role on subjective temporal distance. By manipulating temporal distance, Study 3 tested its causal effects on intergroup outcomes and whether glorification moderated these effects.

The present research contributes to the literature in two main ways. First, by directly comparing victim and perpetrator perspectives after intergroup violence, this work adds to the emerging research on asymmetric responses between conflict parties (for reviews, see Bilali & Vollhardt, 2019; Li & Leidner, 2019). Second, this research is among the first to examine temporal distance in intergroup contexts (see also Greenaway et al., 2012; Peetz et al., 2010) and the first to experimentally investigate victim group members’ temporal perceptions.

## Study 1

The main purpose of Study 1 was to examine the role of temporal distance in people’s responses to intergroup violence among both victim and perpetrator groups, in the context of the conflict between Bosniaks and Serbs. As one of the most destructive conflicts of the late 20th century, the Bosnian War resulted in an estimated 100,000 casualties of combatants and civilians. Although both sides have committed acts of violence, historical consensus maintains that Serbs were the primary perpetrators and Bosniaks the primary victims of the conflict. Against this backdrop, Study 1 examined both Serbs’ and Bosniaks’ subjective perceptions of temporal distance and attitudes toward various issues related to justice and reconciliation.

Study 1 also examined participants’ reactions to episodes of violence either committed or suffered by their own ethnic group. In other words, within each ethnic group we experimentally manipulated the ingroup’s victim versus perpetrator role in a specific conflict scenario. Thus, Study 1 explored the ingroup’s role in intergroup violence (victim vs. perpetrator) both experimentally, by highlighting a specific episode of violence, and quasi-experimentally, by using ethnicity (Bosniak vs. Serb) as a proxy for the ingroup’s overall role in the conflict. We hypothesized that among Serbs, more subjective temporal distancing from the Bosnian War should predict less support for justice and conciliatory efforts, as well as less empathy toward Bosniaks. Among Bosniaks, in contrast, more subjective temporal distancing from the war should predict less demand for justice (e.g., punishment of Serb perpetrators), more support for conciliatory efforts, and more empathy toward Serbs. Moreover, Bosniaks should perceive the war as temporally closer than Serbs, again due to their respective victim and perpetrator status in the overall conflict.

While we expected the experimental manipulation of victim versus perpetrator role to produce similar patterns of responses within each ethnic group, the overall victim and perpetrator status of Bosniaks and Serbs might weaken the effects of the role manipulation. The hypothesized relationship between temporal distance and various intergroup outcomes should thus be the strongest when the conflict scenario described in the article is congruent with the overall victim or perpetrator status of the ingroup (i.e., Serbs in the ingroup-perpetrator condition and Bosniaks in the ingroup-victim condition).

### Method

#### Participants

To determine sample size, we followed a general rule of no less than 100 participants per group, which corresponds to high power (>.80) to detect two-way or three-way interactions with an effect size of *f* = .20 (G*power; Faul et al., 2007). We over-sampled to compensate for potential exclusions as a result of data quality screening. Our final sample comprised a total of 570 participants (333 Serbs; 237 Bosniaks).

Serb participants were recruited using a convenience sampling procedure in Serbia. Approximately 100 students from the University of Belgrade served as recruiters and did not fill out the survey themselves. Each recruiter distributed the link to the online survey to three to four respondents using his or her own social networks. We followed a routine data quality screening procedure (e.g., Li et al., 2016, 2020), which resulted in an exclusion of 75 participants (see Supplementary File for a detailed description of the procedure). A total of 258 participants were retained for subsequent data analyses (72% women; age *M* = 26.71, *SD* = 10.37, range = 18–79).

Bosniak participants were also recruited through convenience sampling in Bosnia and Herzegovina. The study was conducted in paper-and-pencil format. We made a deliberate effort to recruit an approximate equal number of male and female participants in each experimental condition. The screening of the data resulted in the exclusion of 18 participants (see Supplementary File). A total of 219 participants were retained for subsequent data analyses (53% women; age *M* = 36.82, *SD* = 11.43, range = 19–68).

#### Procedure

Both Serb and Bosniak participants followed the same study procedure. After consenting to take part in a study on attitudes toward the relations between Serbs and Bosniaks, participants were randomly assigned to read a news article depicting a military operation, led either by Serbs or Bosniaks, in a town in Bosnia and Herzegovina or in Serbia, respectively. During the operation, more than 3,000 civilians were killed and thousands more were injured. In one condition, participants read about Serbs committing war crimes against Bosniak civilians (i.e., ingroup transgression for Serbs and ingroup victimization for Bosniaks). In the other condition, participants read about Bosniaks committing war crimes against Serb civilians (i.e., ingroup victimization for Serbs and ingroup transgression for Bosniaks). The news articles were largely identical across conditions except for the ethnic identities of perpetrators and victims, and the locations of the military operation. After the reading task, participants completed several attention check questions in which they indicated the ethnicities of perpetrators and victims of the violence described in the article. Participants then summarized the article in their own words to demonstrate their understanding of the article. Afterwards, they filled out the following dependent measures in the order described below. All items were measured on 6-point scales (1 = *strongly disagree*; 6 = *strongly agree*) unless noted otherwise. At the end of the study, participants indicated how believable they found the news article (1 = *not at all*; 6 = *very much*),^1^ reported their demographic information, and were fully debriefed.

#### Materials

##### Subjective temporal distance

Following Wohl and McGrath (2007), a single-item measure assessed subjective temporal distance from the event described the article. Participants indicated how distant or close in time they felt to the event by placing a mark on a horizontal line with the left end designated “very distant” and the right end designated “very close.” Because the length of the line in the paper-and-pencil version was different from that in the online version, we used the ratio of the distance between the mark and the right end of the line (“very close”) to the total length of the line as an indicator of subjective temporal distance (0 = *very close*; 100 = *very distant; M* = 46.74, *SD* = 28.59).

##### Attitudes toward justice-related issues

Participants indicated their attitudes toward a variety of issues related to justice, including punishment of the perpetrators, rights for members of the outgroup, domestic policies related to the outgroup, as well as the ICTY.

Demands for retributive justice. Adapted from Leidner et al. (2013), four items measured the extent to which participants supported punishment of the perpetrator group as a way to restore justice (e.g., “To restore justice, Serbia/BiH needs to be punished for its military’s actions described in the news article”; α = .91, *M* = 3.96, *SD* = 1.57).

Support for outgroup rights. To measure participants’ attitudes toward outgroup rights, we developed eight items regarding the basic civil, political, and human rights of Bosniaks currently living in Serbia or Serbs currently living in Bosnia and Herzegovina (e.g., “All Bosniaks/Serbs should be entitled to social security and welfare benefits.”). We reverse-scored three items tapping refusal to grant rights to the outgroup, creating a composite score reflecting support for rights of the outgroup members (α = .82, *M* = 4.51, *SD* = 0.98).

Support for policies toward the outgroup. Nine items measured the extent to which participants supported pro- and anti-outgroup policies, again regarding members of the outgroup currently living in participants’ own country (e.g., “It should be allowed to Bosniaks/Serbs, if they wish, to have dual citizenship.”). Anti-outgroup policy items were reverse-scored (α = .82, *M* = 3.78, *SD* = 1.05).

Attitudes toward the ICTY. A single-item measure was included to assess participants’ general attitudes toward the ICTY (“What is your attitudes toward the ICTY in general?”). This item was directly taken from the “Attitudes toward the International Criminal Tribunal for the Former Yugoslavia (ICTY)” survey series, and was measured on a 4-point scale (1 = *extremely negative*; 4 = *extremely positive; M* = 2.29, *SD* = 0.78). We kept the item in its original format because these data were also used in a different project, where we compared our participants’ responses to this particular question with Serbs’ responses in the survey series in previous years.

##### Willingness to reconcile

To capture participants’ willingness to reconcile with members of the outgroup, we assessed the extent to which participants supported reconciliation at both the state and the personal level.

State-level reconciliation. Adapted from Wenzel and Okimoto (2010), five items measured the extent to which participants supported their ingroup’s effort to promote reconciliation with the outgroup at the state level (e.g., “Serbia/BiH should try to do its part to promote reconciliation with BiH/Serbia”; α = .88, *M* = 4.57, *SD* = 1.16).

Personal-level reconciliation. Four items measured participants’ willingness to personally engage with the people and culture of the outgroup (e.g., “I would like to be friends with a Bosniak/Serb”; α = .89, *M* = 4.25, *SD* = 1.31).

##### Empathy

Adapted from Batson et al. (1987), four items measured the extent to which participants felt empathy for the outgroup (e.g., “I feel compassion for the Bosniaks/Serbs”; α = .92, *M* = 2.76, *SD* = 1.46).

### Results

Table 1 displays the bivariate correlations between variables for Bosniaks in the victim condition and Serbs in the perpetrator condition (i.e., “congruent” conditions).

**Table 1.** Bivariate Correlations by Ethnicity (Study 1).

Measure	Bosniaks/victim condition							Serbs/perpetrator condition						
	1	2	3	4	5	6	7	1	2	3	4	5	6	7
1. Temporal distance														
2. Retributive justice	−.208*							−.192*						
3. Outgroup rights	.163^†^	−.115						−.098	.330***					
4. Pro-outgroup policies	.172^†^	−.294**	.591***					−.152	.333***	.702***				
5. Attitudes toward ICTY	.048	−.023	.205*	.099				−.116	.253**	.252**	.356***			
6. State-level reconciliation	.270**	−.231*	.652***	.534***	.118			−.059	.302**	.642***	.643***	.243**		
7. Personal-level reconciliation	.159^†^	−.239*	.604***	.546***	.127	.692***		−.107	.348***	.657***	.649***	.245**	.732***	
8. Empathy	.250	−.353***	.320***	.410***	−.052	.393***	.391***	−.179^†^	.354***	.616***	.560***	.393***	.583***	.584***

† *p* < .100. *p < .050. **p < .010. ***p < .001.

#### Effects of ingroup role on subjective temporal distance

To assess the joint effects of experimental and quasi-experimental ingroup role (victim vs. perpetrator) on subjective temporal distance, we submitted subjective distance as a dependent variable (DV) to a moderated regression analysis using the general linear model (GLM) procedure in SAS 9.4. In the analysis, ethnicity (Bosniak vs. Serb) and the experimental manipulation (and their interaction) were entered as independent variables (IVs).

The analysis yielded a significant main effect of the quasi-experimental factor on *subjective temporal distance, F*(1, 439) = 24.14, *p* < .001, $\eta_{p}^{2}$ = .05 (lower confidence interval [LCI] = .02, upper confidence interval [UCI] = .09), such that as predicted, Bosniaks perceived the events described in the article as less temporally distant (i.e., temporally closer; *M* = 40.16, *SD* = 28.49) than Serbs (*M* = 53.17, *SD* = 27.24). The experimental factor did not significantly affect participants’ subjective temporal distance (*M*_victim_ *=* 47.82, *M*_perpetrator_ *=* 45.63), *F*(1, 439) = .87, *p* = .351, $\eta_{p}^{2}$ < .01 (LCI < .001, UCI = .01), nor did the interaction, *F*(1, 439) = 2.03, *p* = .155, $\eta_{p}^{2}$ < .001 (LCI < .01, UCI = .02).

We also conducted the same analyses with attitudes toward justice-related issues, willingness to reconcile, and empathy as DVs. The results were consistent with participants’ perceptions of temporal distance: compared to Serbs, Bosniaks reported more demands for retributive justice, less support for outgroup rights and pro-outgroup policies, more support for the ICTY, and more empathy, and they were less willing to reconcile at both state and personal levels (see Table 2 and Supplementary Analysis 2).

**Table 2.** Effects of Ethnicity, Ingroup Role, and Their Interaction on Dependent Variables (Study 1).

DV	Ethnicity		Ingroup role		Ethnicity × ingroup role interaction	
	F	*p*	F	*p*	F	*p*
Temporal distance	**24.14**	**<.001**	0.87	.351	2.03	.155
Retributive justice	**34.05**	**<.001**	**177.69**	**<.001**	**37.57**	**<.001**
Outgroup rights	**5.50**	.**020**	**8.28**	.**004**	1.14	.287
Pro-outgroup policies	**132.54**	**<.001**	**16.54**	**<.001**	0.15	.698
Attitudes toward ICTY	**129.76**	**<.001**	0.09	.762	0.16	.691
State-level reconciliation	**49.11**	**<.001**	**25.67**	**<.001**	**4.18**	.**042**
Personal-level reconciliation	**59.32**	**<.001**	**13.81**	**<.001**	2.89	.090
Empathy	**51.40**	**<.001**	**175.44**	**<.001**	**4.53**	.**034**

*p* values ≤ .050 and corresponding *F* values are in boldface.

#### Implications of temporal distance

To test whether subjective temporal distance has differential relationships with attitudes toward justice and reconciliation, as well as empathy among victim and perpetrator groups, we conducted a fully unconstrained multigroup path analysis to compare model fit among the four different groups (i.e., Bosniak/victim, Bosniak/perpetrator, Serb/victim, Serb/perpetrator). Due to the strong correlation between state- and personal-level reconciliation (*r* = .75) and participants’ similar responses to both scales, we created a new variable using the composite score of the two reconciliation scales (α = .82), tapping participants’ general willingness to reconcile. For the same reason, support for outgroup rights and policies toward the outgroup (*r* = .64) were also combined, creating a new variable tapping participants’ support for pro-outgroup policies on various issues (α = .88). The results reported below thus used the new composite scores.^2^

In the path model, subjective temporal distance was entered as an exogenous variable, and demand for retributive justice, support for pro-outgroup policies, attitudes toward the ICTY, willingness to reconcile, and empathy as endogenous outcome variables.^3^ Because the five outcome variables were closely related, their error terms were allowed to freely covary. The overall model, with all parameters freely estimated in the four groups, provided an adequate fit to the data, χ^2^(4) = 16.42, *p* = .003, comparative fit index (*CFI*) = .98, standardized root mean squared residual (*SRMR*) = .06, Goodness of Fit Index (*GFI*) = .99, Normed Fit Index (*NFI*) = .98. The model had acceptable fit for the data in each subgroup.

**Figure 2.**
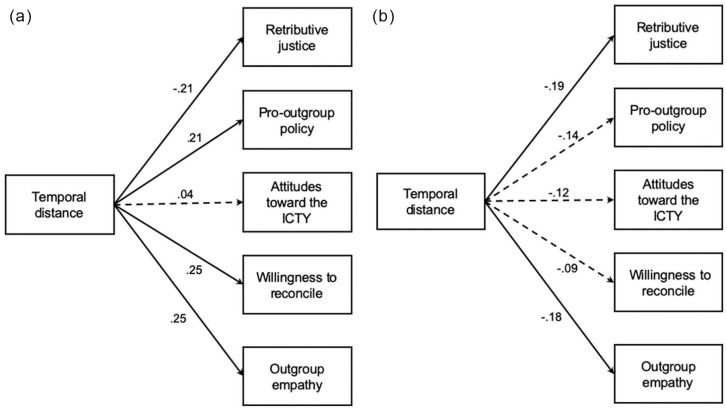
Statistical models depicting the effects of subjective temporal distance on intergroup outcomes among (A) Bosniaks in the ingroup-victim condition and (B) Serbs in the ingroup-perpetrator condition. Solid paths were significant; dashed paths were not (Study 1).

Among Bosniaks in the ingroup-victim condition (SRMR = .04, GFI = .99, NFI = .98; Figure 2A), temporal distancing from the war predicted demands for retributive justice negatively, and support for pro-outgroup policies, willingness to reconcile, and outgroup empathy positively. Subjective temporal distance was not significantly associated with attitudes toward the ICTY. Among Serbs in the ingroup-perpetrator condition (SRMR = .08, GFI = .98, NFI = .96; Figure 2B), in contrast, temporal distancing from the war negatively predicted demands for retributive justice and empathy toward the outgroup. It did not, however, significantly predict any of the other outcome variables. Importantly, the two subgroups differed significantly on the relations between subjective temporal distance on one hand, and support for pro-outgroup policies, reconciliation, and empathy on the other, *ts* > 2.26, *ps <* .011. Although the model also fit the data well in the other two subgroups (Bosniak/perpetrator: SRMR = .02, GFI = .99, NFI = .98; Serb/victim: SRMR = .07, GFI = .98, NFI = .97), subjective temporal distance did not significantly predict any of the intergroup outcomes, βs < .14, *ps* > .130.

### Discussion

Study 1 provided preliminary support for the main hypotheses that temporal distance would be differentially associated with attitudes toward justice and reconciliation, as well as outgroup empathy, among victim and perpetrator group members. Among Bosniaks in the victim condition, subjective temporal distance predicted more positive and conciliatory intergroup attitudes (i.e., less demands for retributive justice, more support for pro-outgroup policies, more willingness to reconcile, and more outgroup empathy). In contrast, among Serbs in the perpetrator condition, subjective temporal distance predicted less conciliatory attitudes (i.e., less support for retributive justice and less outgroup empathy). Although subjective temporal distance was not significantly associated with the other intergroup outcomes among this subgroup, its relationships with support for pro-outgroup policies and reconciliation were significantly different from those among Bosniaks in the victim condition. These significant effects supported our main argument that temporal distancing should have differential implications for intergroup attitudes among victim and perpetrator groups. Importantly, these differential associations were only significant when the conflict scenario described in the article was congruent with participants’ ingroup’s overall victim or perpetrator status in the Bosnian War. This finding suggests that reminders of past episodes of intergroup violence alone may not lead to the observed effects when the ingroup’s role in those past episodes of violence contradicts its role in the broader conflict. Study 1 also lent partial support to the hypothesis that victim group members would perceive the intergroup transgression as temporally closer than perpetrator group members. While the experimental factor did not affect participants’ perceived temporal distance, the quasi-experimental factor did, with Bosniaks in general perceiving the war as temporally closer than Serbs.

Given that both Bosniaks and Serbs were well aware of their group’s role in the broader conflict, participants may not have internalized the manipulated victim vs. perpetrator identity. Indeed, Bosniak participants found the article less believable when their ingroup was portrayed as the perpetrator rather than the victim (Supplementary Analysis 1). It was therefore not surprising that the ingroup role manipulation had rather limited effects on participants’ perceived temporal distance and intergroup attitudes. It should also be noted that the Bosnian War took place on Bosnian, not Serbian, soil. As a result, Serb participants might have felt temporally further away from the conflict than Bosniaks, not only because Serbs were the main perpetrators, but also because the violence was geographically distant and they did not experience violence firsthand. Despite these drawbacks of the quasi-experimental method and the nature of field experiments, Study 1 allowed us to explore the research questions in a real conflict context with participants from both sides of the conflict. Moreover, most participants in both samples had personal ties to the Bosnian War. Thus, the methodological approach of Study 1 enhanced the ecological validity of the findings.

## Study 2

The main goals of Study 2 were twofold. First, it aimed to provide a conceptual replication of Study 1 in a different intergroup context where participants did not have a strong preexisting victim or perpetrator identity—the conflict between the United States and Iran. Moreover, it only employed American participants and experimentally manipulated the victim or perpetrator role of the United States, and was thus not subject to the limitations of quasi-experimental designs. Second, it further examined the effect of the ingroup’s victim versus perpetrator role on subjective temporal distance by (a) using a more elaborate rather than the single-item measure of temporal distance, and (b) investigating the hypothesized moderator of this effect: ingroup glorification.

### Method

#### Participants

The sample consisted of 276 American participants recruited through Amazon’s Mechanical Turk (MTurk). Our routine data screening resulted in an exclusion of 50 participants. 226 participants were retained for subsequent data analyses (60% women; age *M* = 37.84, *SD* = 11.39, range = 18–71).

#### Procedure

Participants followed a similar procedure as in Study 1. First, participants were randomly assigned to read a fictitious, but allegedly real, New York Times article depicting cases of prisoner abuse in a secret prison at the Afghan–Iranian border. In the ingroup-victimization condition, participants read about Iranian soldiers capturing and torturing American civilians, whereas in the ingroup transgression condition participants read about American soldiers capturing and torturing Iranian civilians. The news articles were identical across conditions except for the names and nationalities of perpetrators and victims. After the reading task, participants completed several attention check questions and summarized the news article. Then they filled out the following measures in the order outlined below. All items were measured on 9-point visual analog scales (1 = *strongly disagree*; 9 = *strongly agree*). In addition to retributive aspects of justice, Study 2 also measured demands for restorative aspects of justice, including apology, financial compensation, and efforts to reaffirm shared values between the two groups (e.g., Okimoto et al., 2009). Because Study 2 was situated in an entirely different intergroup context, the other justice-related measures in Study 1 (i.e., support for the rights of and policies toward outgroup members living in the ingroup’s country) were not included in this study. As an additional manipulation check, Study 2 also included a measure directly assessing participants’ perceived victim and perpetrator identity toward the end of the survey. As in Study 1, participants also indicated how believable they found the news article (1 = *not at all*; 9 = *very much*).^4^

#### Materials

##### Subjective temporal distance

Participants indicated how distant or close they felt to the events portrayed in the news article on four different scales ranging from (a) “feel very distant in time” to “feel very close in time”; (b) “feel like a long time ago” to “feel like yesterday”; (c) “feel very far from today” to “feel very close to today”; (d) “happened a long time ago” to “just happened.” A composite score with the average of the four items was created to capture perceived temporal distance (1 = *very close*; 9 = *very distant;* α = .96, *M* = 4.52, *SD* = 1.96).

##### Demands for justice

*Retributive justice* was measured using the same five items as in Study 1, adapted to the U.S.–Iran conflict (α = .93, *M* = 6.15, *SD* = 1.90). *Restorative justice* was measured by five items tapping apologetic behavior, financial reparation, and reaffirmation of shared values as ways to restore justice (e.g., “The U.S./Iranian government should offer sincere apologies to the Iranian/American victims and their families”; “To restore justice, the U.S. and Iran need to agree on rules of a peaceful world”; α = .82, *M* = 6.85, *SD* = 1.50).

##### State- and personal-level reconciliation

Support for state- and personal-level reconciliation was measured with the same items as in Study 1, adapted to the U.S.–Iran conflict. We also created a new variable for willingness to reconcile in general, encompassing both state- and personal-level reconciliation.^5^

##### Empathy

Empathy for the outgroup members was measured using the same items as in Study 1, adapted to the U.S.–Iran conflict (α = .94, *M* = 4.45, *SD* = 2.22).

##### Ingroup attachment and glorification

Adapted from Roccas et al. (2006), *attachment* was measured with eight statements tapping the importance of the United States to participants’ identity and their commitment to the United States (e.g., “Being American is an important part of my identity”; α = .95, *M* = 6.78, *SD* = 1.80). *Glorification* was measured with eight statements tapping participants’ belief in American superiority over other countries, and their deference to American authorities (e.g., “The U.S. is better than other nations in all respects”; α = .90, *M* = 5.16, *SD* = 1.81). Following others (e.g., Leidner et al., 2010; Li et al., 2016, 2018), attachment and glorification were measured at the end of the study to avoid raising participants’ suspicion about the goal of the study.

### Results

Table 3 displays bivariate correlations between variables by condition.

**Table 3.** Bivariate Correlations by Ingroup Role (Study 2).

Measure	Victim						Perpetrator					
	1	2	3	4	5	6	1	2	3	4	5	6
1. Temporal distance												
2. Retributive justice	−.176^†^						−.335***					
3. Restorative justice	−.083	.627***					−.382***	.667***				
4. Willingness to reconcile	.071	−.624***	−.315***				−.306***	.476***	.660***			
5. Glorification	−.224*	.579***	.455**	−.593***			.214*	−.393***	−.326***	−.402***		
6. Attachment	−.223*	.510***	.494*	−.420***	.726***		.194*	−.283**	−.175^†^	−.270**	.716***	
7. Empathy	.088	−.655***	−.433***	.768***	−.596***	−.448***	−.421***	.674***	.734***	.691***	−.354***	−.228**

† *p* < .100. *p < .050. **p < .010. ***p < .001.

#### Manipulation check

The ingroup role manipulation successfully increased participants’ perceived victim or perpetrator status of the United States in its conflict with Iran (see Supplementary Analysis 3).

#### Glorification motivates temporal distancing

Neither attachment nor glorification was affected by condition (see Supplementary Analysis 4), thus allowing us to use them, together with condition, as continuous IVs in the subsequent GLMs.

##### Subjective temporal distance

As predicted, the analysis yielded a significant interaction between ingroup role and glorification on subjective temporal distance, *F*(1, 221) = 11.11, *p* = .001, $\eta_{p}^{2}$ = .04 (LCI = .01, UCI = .10). Analyses of simple effects indicated that participants who strongly glorified the United States (i.e., 1 *SD* above the mean) perceived the prisoner abuse as temporally closer when their ingroup was portrayed as the victim (*M* = 4.12) rather than the perpetrator (*M* = 5.05), *t*(221) = 2.56, *p* = .011. In contrast, low glorifiers (i.e., 1 *SD* below the mean) exhibited the opposite pattern, perceiving the prisoner abuse as more distant when their ingroup was portrayed as the victim (*M* = 4.95) rather than the perpetrator (*M* = 4.17), *t*(221) = −2.13, *p* = .034. Looking at the same two-way interaction from a different angle, glorification was somewhat negatively associated with perceived temporal distance when the ingroup was portrayed as the victim, β = −.21, *t*(221) = −1.82, *p* = .070. By contrast, it was positively associated with temporal distance when the ingroup was portrayed as the perpetrator, β = .22, *t*(221) = 1.97, *p* = .050. None of the main effects reached significance, *Fs*(1, 221) < .09, *ps* > .750, η_p_^2^s < .01 (LCIs < .001, UCIs = .01).

As in Study 1, we also conducted the same GLM analyses with demands for retributive and restorative justice, willingness to reconcile, and empathy as DVs. The results were consistent with participants’ perceptions of temporal distance, especially for high glorifiers. When faced with ingroup victimization (as opposed to perpetration), high glorifiers reported more demands for justice, less willingness to reconcile, and less empathy (see Table 4 and Supplementary Analysis 5).

**Table 4.** Effects of Glorification, Ingroup Role, and Their Interaction on Dependent Variables (Study 2).

DV	Glorification		Ingroup role		Glorification × .ingroup role interaction	
	F	*p*	F	*p*	F	*p*
Temporal distance	0.01	.938	0.08	.782	**11.11**	.**001**
Retributive justice	0.03	.861	0.60	.440	**64.37**	**<.001**
Restorative justice	2.22	.138	1.09	.298	**38.46**	**<.001**
Willingness to reconcile	**39.17**	**<.001**	**6.92**	.**009**	**3.89**	.**050**
Empathy	**32.63**	**<.001**	**66.14**	**<.001**	**4.61**	.**033**

*p* ≤ .050 and corresponding *F* values are in boldface.

#### Implications of temporal distance

**Figure 3.**
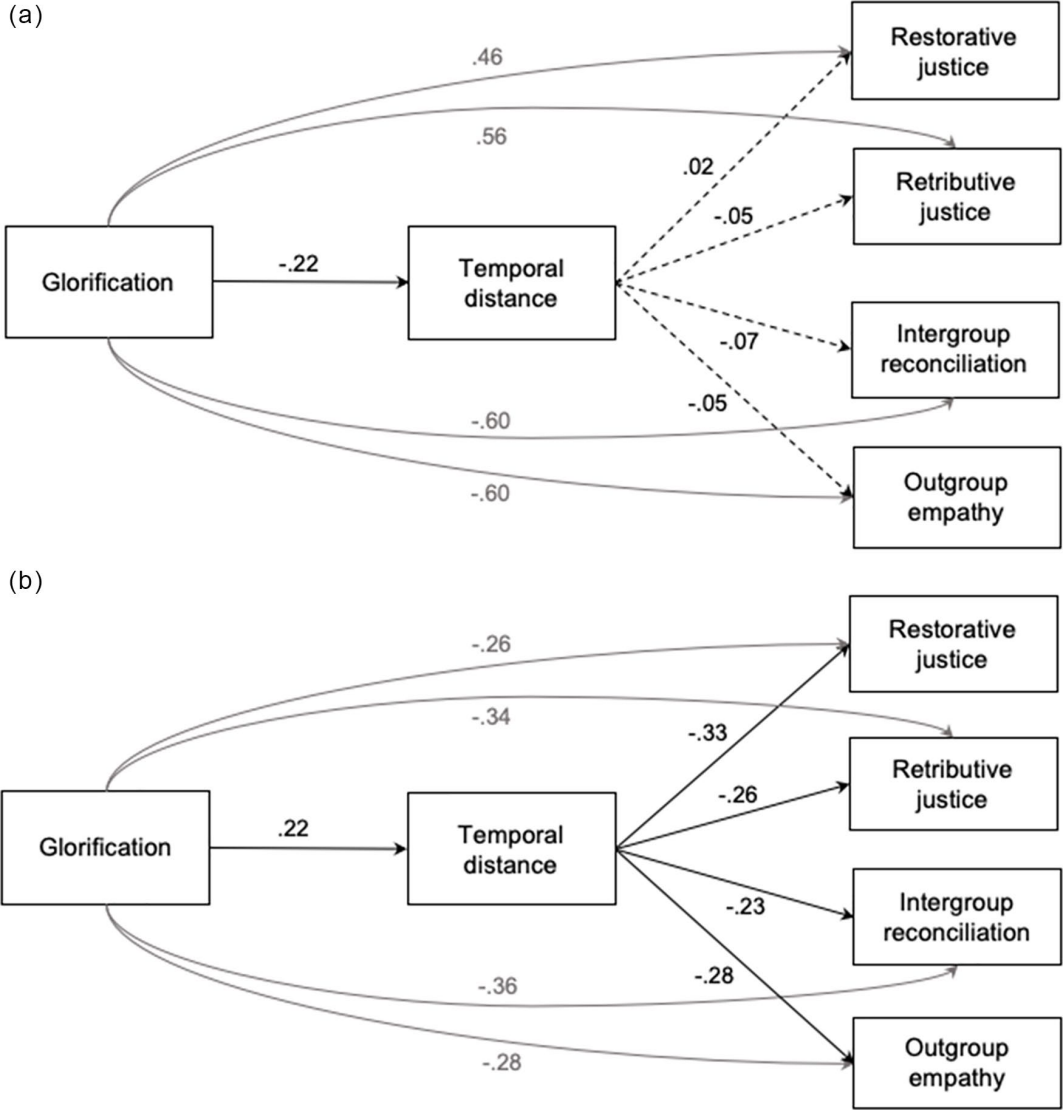
Statistical models depicting the effects of ingroup glorification on intergroup outcomes in (A) the *ingroup-victim condition* and (B) the *ingroup-perpetrator condition*. Paths displayed in black were central to the hypotheses; paths displayed in gray were not. Solid paths were significant; dashed paths were not (Study 2).

To examine whether temporal distance (motivated by glorification) differentially predicted intergroup outcomes among victim and perpetrator groups, we again conducted a fully unconstrained multigroup path analysis to compare model fit between the two experimental conditions. Glorification was entered as an exogenous variable, subjective temporal distance as the mediator, demand for (retributive and restorative) justice, willingness to reconcile, and empathy toward the outgroup as endogenous outcome variables, controlling for attachment as another exogenous variable. To be consistent with the GLMs, the path model also included direct paths from glorification to justice demands, willingness to reconcile, and empathy. Because retributive and restorative justice are two closely related constructs, their error terms were allowed to freely covary, and so were the error terms of retributive and restorative justice, reconciliation, and empathy. The statistical models for the ingroup victim and perpetrator conditions are depicted with standardized path coefficients in Figures 3A and 3B.

The overall model with all parameters freely estimated in the two groups provided an excellent fit to the data, χ^2^(11) = 12.42, *p* = .333, CFI = 1.00, SRMR = .03, GFI = .98, NFI = .98. The model also had adequate fit for the data in each subgroup (victim: SRMR = .04, GFI = .98, NFI = .98; perpetrator: SRMR = .02, GFI = .99, NFI = .99). However, the fit comparison between the two subgroups also revealed that the ingroup-victim condition contributed more to the overall chi-square (78%) than the ingroup-perpetrator condition (22%), suggesting that the model fit for the perpetrator condition was better than that for the victim condition.

In the ingroup-victim condition (Figure 3A), glorification negatively predicted temporal distancing. However, temporal distance did not significantly predict any of the downstream intergroup outcomes. In addition, glorification also predicted willingness to reconcile and outgroup empathy negatively, and demands for retributive and restorative justice positively. In the ingroup-perpetrator condition (Figure 3B), in contrast, glorification predicted temporal distancing positively. Temporal distance, in turn, predicted demands for both retributive and restorative justice negatively, as well as willingness to reconcile and outgroup empathy negatively. In addition, glorification negatively predicted all four outcome variables.

The multigroup path analysis further tested the indirect effects of glorification on justice demands and willingness to reconcile via subjective temporal distance. The analyses revealed indirect effects of glorification on all four outcome variables, two of them significant and two marginally significant, in the ingroup-perpetrator condition (retributive justice: β = −.06, *p =* .061; restorative justice: β = −.07, *p =* .045; reconciliation: β = −.05, *p =* .076; empathy: β = −.07, *p =* .038), but not in the ingroup-victim condition, *ps* > .400. Although the indirect effects in the ingroup-victim condition were not significantly different from zero, they were (marginally) different from those in the ingroup-perpetrator condition, *t*s < −1.70, *ps* < .086, thus supporting the main hypothesis that temporal distance (in conjunction with glorification) plays different roles in predicting intergroup outcomes among victim and perpetrator group members.

### Discussion

Study 2 showed that victim and perpetrator group members indeed had differential subjective perceptions of temporal distance, but the direction of the difference depended on the extent to which they glorified their respective group. Whereas high glorifiers perceived past intergroup transgressions as temporally closer when the ingroup was the victim than when it was the perpetrator, low glorifiers exhibited the exact opposite pattern, perceiving the transgressions as more distant when the ingroup was the victim than when it was the perpetrator. To unpack this interaction effects differently, whereas glorification motivated less temporal distancing among victim group members, it motivated more temporal distancing among perpetrator group members. The absence of a main effect of ingroup role on subjective temporal distance might be attributed to the relatively low relevance of the conflict to the American participants. In Study 1, both Serb and Bosniak participants had personal experiences with the war, and thus even low glorifiers’ time perceptions might have been driven by ingroup-defensive motivations. In contrast, the fictitious incidents of prisoner abuse in the current study were rather far removed from American participants’ everyday lives, making it possible for low glorifiers to react in an ingroup-critical and even pro-outgroup manner.

The multigroup path analysis lent partial support for the hypothesis that temporal distance plays differential roles in perpetrator and victim groups’ responses to intergroup violence. Consistent with Study 1, subjective temporal distancing from the ingroup’s past transgressions (motivated by glorification) predicted perpetrator group members’ reduced support for retributive justice and empathy toward outgroup victims. In addition and as expected, subjective temporal distance also predicted perpetrator group members’ reduced support for restorative justice and willingness to reconcile with the victim group. However, because subjective temporal distance did not significantly predict intergroup outcomes among victim group members, the current study offered only partial evidence for the hypothesized downstream implications of temporal distancing. While it remains unclear why temporal distance did not predict intergroup outcomes among victim group members, we can still safely conclude that perceptions of temporal distance from past intergroup conflict have divergent implications for attitudes toward justice and reconciliation among victim and perpetrator groups.

So far, Studies 1 and 2 demonstrated that temporal distance has differential implications for attitudes toward justice and reconciliation, as well as outgroup empathy, between victim and perpetrator groups. Nonetheless, as the evidence was only correlational, we were unable to establish a causal relationship between temporal distance and intergroup outcomes. Although Study 2 confirmed the hypothesis that subjective temporal distancing is motivated by ingroup glorification, it did not examine whether the differential implications of temporal distance for intergroup outcomes among victim and perpetrator groups also depended on glorification. This limitation was due to the focus on subjective perceptions of temporal distance as a motivated cognition, and the fact that it was statistically impossible to test the complete conceptual model where glorification both served as a predictor of subjective temporal distance and a moderator of the relationship between temporal distance and downstream intergroup outcomes. Study 3 thus addressed these limitations.

## Study 3

Study 3’s main aim was to establish the causal effect of temporal distance on intergroup attitudes. In addition, it aimed to examine whether glorification moderated the effects of temporal distance on victims’ and perpetrators’ attitudes toward justice and reconciliation, and outgroup empathy. To this end, we experimentally manipulated temporal distance by informing participants of an intergroup transgression that took place either in the recent or distant past. As in Studies 1 and 2, Study 3 also experimentally manipulated the victim and perpetrator role of participants’ ingroup in the conflict. We hypothesized that temporal distance would have differential effects on intergroup outcomes depending on whether participants belonged to the victim or perpetrator group and their glorification of the ingroup. Given that the study was again conducted in the context of the U.S.–Iran conflict, we expected high glorifiers to be ingroup-defensive and low glorifiers to be ingroup-critical and pro-outgroup. In response to a temporally distant (vs. recent) transgression, high glorifiers should be particularly likely to show weaker support for justice and reconciliation and reduced empathy when the ingroup was the perpetrator. Due to their strong ingroup-defensive motivation, however, temporally distancing them from past victimization might not be effective in inducing positive intergroup attitudes. Low glorifiers, by contrast, should be particularly likely to show more positive intergroup attitudes in response to a temporally distant (vs. recent) incident of ingroup victimization. Due to their strong ingroup-critical tendencies, however, temporally distancing them from their ingroup’s past transgression might not serve the same defensive function as it does for high glorifiers.

### Method

#### Participants

The sample consisted of 604 participants recruited through MTurk. Our data screening followed the same procedure as in Study 2. As a result, 444 participants were retained in the subsequent data analyses (59% women; age *M* = 38.59, *SD* = 12.39, range = 19–98).

#### Procedure and materials

Study 3 employed a 2 (temporal distance: distant vs. close) × 2 (ingroup role: victim vs. perpetrator) experimental design. In the temporally distant condition, participants learned from a fictitious newspaper article that Iran’s 1979 Islamic Revolution caused a rupture of U.S.–Iran relations, and that a number of Iranian or American workers were detained and tortured in 1980 at a secret prison at the Iran–Afghan border that was either under the control of the U.S. army or the Iranian Revolutionary Guards (depending on victim vs. perpetrator condition). In the temporally close condition, participants learned that tensions between the United States and Iran had mounted due to Iran’s nuclear program, and that Iranian or Americans were detained and tortured in 2011 at a secret prison. To strengthen the temporal distance manipulation, the news article also displayed a visual timeline that marked 1980 or 2011 (otherwise being identical) in the distant and recent condition, respectively.

As a manipulation check, participants in all four conditions then reported their perceived temporal distance to the event described in the article using the same four-item measure as in Study 2. Afterwards, they completed the same measures as in Study 2.

### Results

Table 5 displays bivariate correlations between variables by condition.

**Table 5.** Bivariate Correlations by Ingroup Role (Study 3).

Measure	Victim						Perpetrator					
	1	2	3	4	5	6	1	2	3	4	5	6
1. Temporal distance												
2. Retributive justice	−.153*						−.361***					
3. Restorative justice	−.151*	.469***					−.270***	.665***				
4. Willingness to reconcile	.070	−.458***	−.084				−.244***	.562***	.607***			
5. Glorification	−.035	.309***	.099	−.502***			.198**	−.479***	−.394***	−.613***		
6. Attachment	−.010	.250***	.180**	−.314***	.747***		.071	−.293**	−.156*	−.291***	.662***	
7. Empathy	.139*	−.432***	−.147*	.718***	−.459***	−.361***	−.261***	.630***	.715***	.731***	−.484***	−.206**

* *p* < .050. **p < .010. ***p < .001.

#### Manipulation checks

Analyses revealed that the temporal distance manipulation successfully induced perceptions of past intergroup violence as either temporally close or distant. The ingroup role manipulation again successfully increased perceived victim or perpetrator status of the United States (see Supplementary Analyses 6 and 7).

#### The effects of temporal distance on intergroup outcomes

We first conducted GLMs to test the joint effects of ingroup role, (manipulated) temporal distance, and glorification on demands for retributive and restorative justice, willingness to reconcile, and empathy (see Table 6). All analyses controlled for attachment as a covariate. Below we focus on the results of the hypothesized three-way interactions of the ingroup role and temporal distance manipulations by glorification. The remaining effects are reported in Supplementary Analysis 8.

**Table 6.** Effects of Glorification, Ingroup Role, (Manipulated) Temporal Distance (TD), and Their Interactions on DVs (Study 3).

DV	Glorification		Ingroup role		TD		Glorification × ingroup role		Glorification × TD		Ingroup role × TD		Glorification × ingroup role × TD	
	*F*	*p*	*F*	*p*	*F*	*p*	*F*	*p*	*F*	*p*	*F*	*p*	*F*	*p*
Subjective TD	2.75	.098	0.36	.551	**100.51**	**<.001**	**7.26**	.**007**	0.76	.384	0.69	.407	0.53	.466
Retributive justice	**4.19**	.**041**	**28.13**	**<.001**	**42.80**	**<.001**	**78.81**	**<.001**	0.86	.354	0.01	.903	**4.84**	.**028**
Restorative justice	**24.28**	**<.001**	**16.93**	**<.001**	**10.99**	.**001**	**27.02**	**<.001**	0.19	.660	1.01	.316	**6.38**	.**012**
Willingness to reconcile	**151.08**	**<.001**	1.23	.268	3.09	.079	0.73	.393	3.50	.062	**12.87**	**<.001**	0.07	.799
Empathy	**84.83**	**<.001**	**132.83**	**<.001**	0.95	.330	0.37	.541	**5.28**	.**022**	**23.05**	**<.001**	3.13	.078

*p* ≤ .050 and corresponding *F* values are in boldface.

##### Retributive justice

Demands for retributive justice (α = .93, *M* = 5.90, *SD* = 1.90) was submitted as the DV to the GLM as described above. Consistent with the focal hypothesis of this study, the three-way interaction between the role and distance manipulations by glorification was significant, *F*(1, 435) = 4.84, *p* = .028, $\eta_{p}^{2}$ = .01 (LCI < .01, UCI = .03). As predicted, analyses of simple effects revealed that when the ingroup was the victim, low glorifiers demanded less retributive justice when the conflict was distant (*M =* 5.26) rather than close in time (*M =* 6.46), *t*(435) = 3.71, *p* < .001. High glorifiers, in contrast, exhibited the same pattern, but to a lesser degree (*M*_distant_ *=* 6.41, *M*_close_ *=* 7.21), *t*(435) = 2.65, *p* = .008. When the ingroup was the perpetrator, high glorifiers supported significantly less retributive justice when the conflict was distant (*M* = 3.81) rather than close in time (*M* = 5.34), *t*(435) = 4.66, *p* < .001. Low glorifiers, in contrast, exhibited the same pattern, but to a lesser degree (*M*_distant_ *=* 6.17, *M*_close_ *=* 6.71), *t*(435) = 1.79, *p* = .074.

##### Restorative justice

The analysis with demands for restorative justice (*α* = .81, *M* = 6.71, *SD* = 1.54) as the DV again yielded a significant three-way interaction between the role and distance manipulations by glorification, *F*(1, 435) = 6.38, *p* = .012, $\eta_{p}^{2}$ = .01 (LCI < .01, UCI = .04). Consistent with our hypothesis, when the ingroup was the victim, low glorifiers demanded significantly less restorative justice when the conflict was distant (*M* = 6.81) rather than close in time (*M* = 7.41), *t*(435) = 2.13, *p* = .033. High glorifiers, in contrast, did not differ depending on when the ingroup victimization occurred (*M*_distant_ *=* 6.87, *M*_close_ *=* 6.89), *t*(435) = 0.09, *p* = .925. When the ingroup was the perpetrator, high glorifiers supported significantly less restorative justice when the conflict was distant (*M* = 5.11) rather than close in time (*M* = 6.10), *t*(435) = 3.47, *p* = .001. Low glorifiers, in contrast, did not differ depending on when the ingroup transgression occurred (*M*_distant_ *=* 7.19, *M*_close_ *=* 7.36), *t*(435) = 0.67, *p* = .506.

##### Willingness to reconcile

Participants’ willingness to reconcile with the outgroup (*α* = .93, *M* = 5.29, *SD* = 1.74) was not significantly affected by the three-way interaction between ingroup role and temporal distance and glorification, *F*(1, 435) = .07, *p* = .800, $\eta_{p}^{2}$ = .01 (LCI < .01, UCI = .01). The interaction between ingroup role and temporal distance, however, was significant, *F*(1, 435) = 12.87, *p* < .001, $\eta_{p}^{2}$ = .03 (LCI = .01, UCI = .06). Analyses of simple effects indicated that when the ingroup was the victim, participants expressed more willingness to reconcile when the conflict was distant (*M* = 5.56) rather than close in time (*M* = 4.84), *t*(435) = −3.79, *p* < .001. When the ingroup was the perpetrator, in contrast, participants did not differ significantly in their willingness to reconcile depending on when the transgression took place (*M*_distant_ = 5.23, *M*_close_ = 5.47), *t*(435) = 1.29, *p* = .198.

##### Empathy

The analysis with empathy toward the outgroup as the DV (*α* = .94, *M* = 4.23, *SD* = 2.22) revealed a marginally significant three-way interaction between the role and distance manipulations and glorification, *F*(1, 435) = 3.13, *p* = .078, $\eta_{p}^{2}$ = .01 (LCI < .01, UCI = .03). As predicted, analyses of simple effects revealed that when the ingroup was the victim, low glorifiers were significantly more empathic toward the outgroup when the conflict was distant (*M* = 5.12) rather than close in time (*M* = 3.48), *t*(435) = −4.77, *p* < .001. High glorifiers, in contrast, did not differ in their empathy toward the outgroup depending on when the ingroup victimization occurred (*M*_distant_ *=* 2.37, *M*_close_ *=* 2.11), *t*(435) = −0.84, *p* = .402. When the ingroup was the perpetrator, on the contrary, high glorifiers expressed significantly less empathy when the conflict was distant (*M* = 3.68) rather than close in time (*M* = 4.40), *t*(435) = 2.06, *p* = .040. Low glorifiers, in contrast, exhibited the same pattern, but to a lesser degree (*M*_distant_ *=* 6.03, *M*_close_ *=* 6.57), *t*(435) = 1.69, *p* = .092.

### Discussion

Study 3 experimentally manipulated both temporal distance from intergroup conflict and the ingroup’s perpetrator or victim role in the conflict. Consistent with predictions, temporal distance affected victim and perpetrator group members’ responses to intergroup violence differently, and the effects of temporal distance depended on ingroup glorification (except for willingness to reconcile). As time separated people from past intergroup violence, victim group members—especially those who did not glorify the ingroup—became less demanding of both retributive and restorative justice and empathized more with the outgroup. On the contrary, perpetrator group members—especially those who strongly glorified the ingroup—became less supportive of both types of justice and empathized less with the outgroup. Despite the lack of a three-way interaction between ingroup role, temporal distance, and ingroup glorification on willingness to reconcile, the two-way interaction between ingroup role and temporal distance confirmed our hypothesis that victims were more conciliatory when the past suffering was distant rather than close. Such positive effects of temporal distance, by contrast, did not occur for perpetrator group members.

## General Discussion

The present research investigated the role of temporal distance in attitudes toward justice and reconciliation, as well as outgroup empathy after large-scale intergroup violence from the perspectives of both victim and perpetrator groups. In the context of the conflict between Serbs and Bosniaks, Study 1 provided preliminary evidence that temporal distance from intergroup transgressions played different roles in victim and perpetrator group members’ responses to the transgressions. Whereas increased subjective temporal distance predicted more conciliatory attitudes among the victim group, it predicted less support for retributive justice and less outgroup empathy among the perpetrator group. Study 1 further showed that compared with Serbs, Bosniaks perceived the war as temporally closer. Using a mixed (experimental and quasi-experimental) design, Study 1 also illuminated the importance of the preexisting victim or perpetrator status of the ingroup in influencing group members’ responses to specific episodes of intergroup conflict.

Study 2 successfully manipulated the victim and perpetrator role of the United States in its conflict with Iran. Instead of replicating the direct effect of group role on temporal distance observed in Study 1, Study 2 demonstrated that this effect was moderated by ingroup glorification. Whereas high glorifiers perceived ingroup victimization as temporally closer than ingroup transgression, low glorifiers reacted in the opposite manner. Finally, by experimentally manipulating temporal distance, Study 3 showed that temporal distance causally affected support for justice and reconciliation efforts as well as outgroup empathy, and glorification moderated these effects (except for willingness to reconcile). The use of fictitious, but allegedly real, incidents of intergroup violence in Studies 2 and 3 further suggests that temporal perceptions and their downstream effects do not rely on individuals’ actual memories of a past event. Rather, temporal distance can be constructed for a newly discovered event said to take place in the past.

### Temporal Asymmetry Between Victim and Perpetrator Groups

Recent work on intergroup transgressions has identified a number of ways in which victims and perpetrators differ in their perceptions of and responses to injustice (Bilali & Vollhardt, 2019; Li & Leidner, 2019). The present research contributes to this literature by demonstrating the temporal asymmetry between victim and perpetrator groups. In the aftermath of intergroup conflict, victim and perpetrator groups diverge on the temporal dimension in two major ways.

First, temporal distance has different implications for attitudes toward justice and reconciliation between victim and perpetrator groups. As time (objectively or subjectively) distances group members from the conflict, victim group members become more empathic toward the perpetrator group, and more willing to let go of the past and to reconcile (Studies 1 and 3), whereas perpetrator group members become less empathic toward the victim group and less interested in justice and reconciliation (Studies 1–3). Study 3 further demonstrated the moderating role of glorification, such that the “healing” effects of temporal distance on victim group members were more pronounced among low glorifiers, whereas the morally disengaging effects of temporal distance on perpetrator group members were more pronounced among high glorifiers. It is worth noting that increased temporal distance reduced low glorifiers’ demands for both retributive and restorative justice in response to ingroup victimization in a conflict. Given that victims’ endorsement for restorative justice generally leads to support for peaceful conflict resolution (e.g., Leidner et al., 2013), the decrease in restorative justice demands might be seen as a negative rather than positive effect of temporal distance on victims’ intergroup attitudes. However, while this might be true, low glorifying victims’ general tendency to let go of the past and make peace with the outgroup as time passes suggests that their reduced restorative demands more likely reflect an increased willingness to move on from the past conflict rather than reduced interest in the peace process. Moreover, low glorifiers’ average demands for both retributive and restorative justice were in fact very high (above the mid-point of the scales in all four experimental conditions), indicating their general support for (rather than resistance to) justice efforts.

In the second temporal asymmetry, victim and perpetrator group members diverge in their subjective perceptions of temporal distance. Victim group members tend to perceive past injustices as temporally closer than perpetrator group members. Study 2 suggests that this type of temporal asymmetry is particularly salient among people who strongly glorify the ingroup. Overall, these asymmetric responses to intergroup violence, especially those of high glorifiers, can constitute a barrier to an effective pursuit of justice and intergroup reconciliation.

### Implications for Other Dimensions of Psychological Distance

Given that different dimensions of psychological distance (temporal, spatial, social, and hypotheticality) are often interrelated (Trope & Liberman, 2003), the findings of the current research might also generalize beyond temporal distance. For instance, perpetrator group members might also be motivated to perceive the transgression as taking place further away in space than are victim group members. Spatially distancing oneself from past violence might similarly lead to less empathy and less interest in justice among the perpetrator group, and more positive intergroup outcomes among the victim group. Temporal distance, however, bears unique importance in the aftermath of intergroup violence, as the timing of addressing past wrongdoings is a key issue of transitional justice and often subject to heated debates between different parties involved in the conflict.

### Implications for Post-Conflict Peacebuilding

This research sheds light on when might be a relatively ideal window of time to pursue justice and engage in peacebuilding efforts without engendering pejorative reactions from either side of the conflict. Due to the morally disengaging function of temporal distance for perpetrators and victims’ tendency to perceive their past suffering as still close in time, the current findings suggest that, all else being equal, the ideal timing for the pursuit of post-conflict or transitional justice should be sooner rather than later. This conclusion may seem to contradict the belief that the pursuit of criminal justice immediately after conflict would be challenged with strong resistance from perpetrator group members, and therefore obstruct the long-term goal of peace (e.g., Leebaw, 2008). In fact, however, it appears possible that while perpetrator group members tend to react with resistance and even hostility toward early justice efforts, such negative reactions might become even stronger as time distances them from the transgression.

## Supplemental Material

Li_Online_Appendix – Supplemental material for Close or Distant Past? The Role of Temporal Distance in Responses to Intergroup Violence From Victim and Perpetrator Perspectives Click here for additional data file. Supplemental material, Li_Online_Appendix for Close or Distant Past? The Role of Temporal Distance in Responses to Intergroup Violence From Victim and Perpetrator Perspectives by Mengyao Li, Bernhard Leidner, Nebojša Petrović and Nedim Prelic in Personality and Social Psychology Bulletin

Temporal_distance_Supplementary_document_06-12-2020 – Supplemental material for Close or Distant Past? The Role of Temporal Distance in Responses to Intergroup Violence From Victim and Perpetrator Perspectives Click here for additional data file. Supplemental material, Temporal_distance_Supplementary_document_06-12-2020 for Close or Distant Past? The Role of Temporal Distance in Responses to Intergroup Violence From Victim and Perpetrator Perspectives by Mengyao Li, Bernhard Leidner, Nebojša Petrović and Nedim Prelic in Personality and Social Psychology Bulletin

## Notes

1. Serb participants in both experimental conditions and Bosniak participants in the ingroup-victim condition perceived the article relatively believable, scoring above the mid-point of the scale. Nonetheless, both the ingroup role manipulation and ethnicity affected perceived believability of the article (see Supplementary Analysis 1).

2. Using the original scales for the dependent variables (DVs) produced the same patterns of results.

3. In all studies, we also tested an alternative model where empathy was treated as a mediator, explaining the relationship between subjective temporal distance and the other conflict-specific DVs. The results are reported in Supplementary Analysis 9.

4. Participants perceived the article to be relatively believable, scoring above the mid-point of the scale. Participants found the article somewhat more believable when the ingroup was portrayed as the victim (*M* = 6.94) than the perpetrator (*M* = 6.36), *F*(1, 221) = 3.65, *p* = .057.

5. Personal- and state-level reconciliation were again highly correlated (*r* = .71), and an EFA revealed a satisfactory one-factor solution.
