# Supplementary material for: Close or Distant Past? The Role of Temporal Distance in Responses to Intergroup Violence From Victim and Perpetrator Perspectives
Source: Pers Soc Psychol Bull. 2020 Aug 1;47(4):657–72. doi: 10.1177/0146167220945890 (PMC7961862; doi:10.1177/0146167220945890)
Supplement: Li_Online_Appendix – Supplemental material for Close or Distant Past? The Role of Temporal Distance in Responses to Intergroup Violence From Victim and Perpetrator Perspectives [file Li_Online_Appendix.docx]

**Study 1**

**Manipulation Materials: Victim vs. Perpetrator Role Manipulation**

***Ingroup-perpetrator condition:***

**The conflict in the Balkans endured for decades and led to a substantial number of casualties among all sides of the conflict. Many experts agree that during the conflict there were many incidents in which innocent Serbs were badly injured or killed by Bosniaks, as well as many incidents in which innocent Bosniaks were badly injured or killed by Serbs. Nevertheless, now and later on in the survey, you are asked to focus on and think only about the incidents in which Bosniaks were severely harmed or killed by Serbs.** **In this survey, we will first present you with a short newspaper article on the conflict between Serbs and Bosniaks. Later on, we will ask you a set of questions related to that article. So please read this article very carefully and pay close attention to the content, as we will ask you questions about it afterwards.**

**Bosniak Victims of Eastern Bosnia Remembered**

VISEGRAD -- A ceremony dedicated to the Bosniaks killed by Serb forces under Nenad Tanasković’s command will be held in Visegrad on Tuesday. The commemoration for the Bosniak victims in the Visegrad region is organized by the Association of Veterans of Patriotic League of Bosnia and Herzegovina.

Tanasković and his forces occupied the town of Visegrad and the surrounding villages on April 6, 1992. Sixty-nine Bosniak civilians were killed and 70 more were wounded, while 19 are still reported as missing. During the following months, more than 3,000 Bosniaks were killed, a majority of whom were civilians, women and children. Hundreds of surviving children were left either without one or both parents, while thousands of families lost their property. Dozens of primarily Bosniak villages were destroyed.

The commemoration for the Bosniak victims in the region is organized by the Federation of Patriotic League Veteran Association of Bosnia and Herzegovina.

***Ingroup-victim condition:***

**The conflict in the Balkans endured for decades and led to a substantial number of casualties among all sides of the conflict. Many experts agree that during the conflict there were many incidents in which innocent Serbs were badly injured or killed by Bosniaks, as well as many incidents in which innocent Bosniaks were badly injured or killed by Serbs. Nevertheless, now and later on in the survey, you are asked to focus on and think only about the incidents in which Serbs were severely harmed or killed by Bosniaks.** **In this survey, we will first present you with a short newspaper article on the conflict between Serbs and Bosniaks. Later on, we will ask you a set of questions related to that article. So please read this article very carefully and pay close attention to the content, as we will ask you questions about it afterwards.**

**Serb victims of Eastern Bosnia Remembered**

BRATUNAC -- A ceremony dedicated to the Serbs killed by Muslim forces under Naser Orić's command will be held in Bratunac on Tuesday. The commemoration for the Serb victims in the region is organized by the Board for Fostering the Tradition of the Liberation Wars of the government of the Serb Republic.

Orić and his forces occupied the town of Bratunac and the surrounding villages on July 12, 1992. Sixty-nine Serb civilians were killed and 70 more were wounded, while 19 are still reported as missing. During the following months, more than 3,000 Serbs were killed, a majority of whom were civilians, women and children. Hundreds of surviving children were left either without one or both parents, while thousands of families lost their property. Dozens of primarily Serb villages were destroyed.

The commemoration for the Serb victims in the region is organized by the Board for Fostering the Tradition of the Liberation Wars of the government of the Serb Republic.

**[Victim vs. perpetrator role manipulation checks]**

**The remainder of the survey will focus on your perception of the events reported in the newspaper article you have just read. First, we have a few questions regarding your understanding of the newspaper article. For each of the questions below, please choose only ONE answer by checking one of the circles on the left.**

Who were the victims in the events you have just read about in the news report?

- Serbs
- Bosniaks
- Croats
- Both sides were victims and perpetrators
- Do not know

Who were the perpetrators in the events you have just read about in the news report?

- Serbs
- Bosniaks
- Croats
- Both sides were victims and perpetrators
- Do not know

**Next, we would like you to write a brief summary of what you have read about in the beginning of this study. The goal is that a third person who has not read the document will be able to understand its contents by just reading your summary. Please address specifically the content of the conflict and how it was handled afterwards.**

**Measures^[[1]](#footnote-1)^**

**[Subjective Temporal Distance]**

**How distant or close in time do you feel the events you read about in the news article? Please indicate your answer by marking a spot on the horizontal line below.**

Very close

Very distant

**[Empathy toward the Outgroup]^[[2]](#footnote-2)^**

**The following questions will focus on how you feel and think about Bosniaks after reading about the events described in the news article. Please take a moment and think about the events and how they were addressed by a tribunal.**

**As the focus is on your thoughts and feelings, there are no correct or incorrect answers.**

**Please indicate the extent to which you agree or disagree with the following statements.**

1. I feel compassion for the Bosniaks.
2. I feel sympathy for the Bosniaks.
3. I feel warmth for the Bosniaks.
4. I feel close to the Bosniaks.

**[Attitudes toward justice-related issues]**

*Demands for Retributive Justice****^[[3]](#footnote-3)^***

**Now we are interested in how much, in your opinion, further efforts need to be made to restore justice after the conflict described in the news article. Please report what you think about the following possibilities.**

**There are no right or wrong answers. Please indicate the extent to which you agree or disagree with the following statements.**

1. To restore justice, Serbia needs to be punished for its military’s actions described in the news article.
2. For justice to be fully reinstated, Serbia needs to be held accountable for its military actions against Bosniaks.
3. For justice to be fully reinstated, Serbia needs to be punished for what its military has done in the events described in the article.
4. To fully reinstate justice, the Human Rights Court needs to prosecute Serbia for its role in the events described in the news article.

***Support for Rights for the Outgroup^[[4]](#footnote-4)^***

**The following questions will focus on your attitudes toward social and political issues with regards to Bosniaks in Serbia.**

**Please indicate the extent to which you agree or disagree with the following statements.**

1. All Bosniaks in need should be eligible for government subsidy programs (e.g., food, housing, health insurance).
2. All Bosniaks should be entitled to social security and welfare benefits.
3. Serbia should have the right to exile Bosniaks who hold political views that endanger Serbia’s national goals.
4. There are times when Bosniaks should be kept from expressing their opinion.
5. Some Bosniaks should not take part in the Serbian government.
6. Every Bosniak should have the right to run for all levels of political office in Serbia, including president, prime minister, minister of defense, etc..
7. All Bosniaks have the right to just and reasonable pay for work performed.
8. Bosniaks should have the right to express their religious views even in the public sphere.

***Support for Policies toward the Outgroup^[[5]](#footnote-5)^***

**The following questions will focus on your attitudes toward specific policies with regards to Bosniaks in Serbia.**

**Please indicate the extent to which you agree or disagree with the following statements.**

1. Every vote of members of my nation in elections would have to be worth at least twice as much as the voice of Bosniaks.
2. Mixed marriages between Bosniaks and Serbs is not always a good idea.
3. Bosniaks should be considered as our brothers and sisters.
4. It must not be allowed to Bosniaks to have a TV and radio stations.
5. Bosniaks must not be allowed public appearance that glorifies their own national history.
6. It should be allowed to Bosniaks, if they wish, to have dual citizenship.
7. Bosniaks should have their own schools in their own language.
8. Bosniaks’ children should be allowed to learn their own national history, culture and literature according to programs from Sarajevo.
9. Bosniaks in Serbia should be more politically and economically connected with Bosnia and Herzegovina.

***Attitudes toward the ICTY***

What is your attitude on ICTY in general?

- Extremely negative
- Mainly negative
- Mainly positive
- Extremely positive

**[Willingness to Reconcile]^[[6]](#footnote-6)^**

***State-level Reconciliation***

**Now we are interested in how you envision the future relationship between Serbia and BH. Again, please provide your personal opinion. There are no right or wrong answers.**

**Please indicate the extent to which you agree or disagree with the following statements.**

1. Serbia should try to do its part to promote reconciliation with BiH.
2. Serbia should express good will toward BiH.
3. Serbia should do its part to improve the atmosphere between Serbia and BiH.
4. Serbia should keep its distance from BiH.
5. Serbia should not trust BiH.

***Personal-level Reconciliation***

**Now we are interested in what you think Serbs should do now.**

**There are no right or wrong answers. Please indicate the extent to which you agree or disagree with the following statements.**

1. Serbs should be allowed to move on with their lives.
2. Serbs should be allowed to turn their attention to other societal issues and international affairs that concern Serbia.
3. Serbs should be allowed to let go of what happened during the events in 1992.
4. Serbs should be allowed to focus more on moving forward as a nation.

**[Believability]**

How believable did you find the news article that you read at the beginning of this survey?

**(Not at all --- very much)**

**Studies 2 & 3**

**Manipulation Materials**

**Study 2: Victim vs. Perpetrator Role Manipulation**

***Ingroup-perpetrator condition:***

**Investigation Revealed American Abuse of Iranian Prisoners**

A recent investigation conducted by the Committee for Justice in the Middle East (CJME), revealed cases of serious abuse of Iranian prisoners at a secret prison at the Iran-Afghan border that is under the control of the United States Armed Forces.

In recent years, tensions have mounted between the United States and Iran due to increasing concerns about Iran’s nuclear program. In 2011, the two countries elevated the belligerent tone between them over an Iranian vow to close the Strait of Hormuz, a vital Middle East waterway for oil tanker traffic, if the U.S. attempted to make good on its threat to stifle Iran’s petroleum exports.

With the escalation of the conflict between Iran and the United States, a number of Iranians working at the border were detained and interrogated by the U.S. military.

Brigadier General Adam McLaughlin, formerly in charge of the prison, provided information in an interview with a CJME official investigating the abuses at the prison. The transcript of this May 2012 interview was among hundreds of pages of papers obtained by CJME.

The investigation provides detailed descriptions of three cases of severe maltreatment of Iranian detainees at the prison.

One of the detainees was placed in solitary confinement with his eyes blindfolded and his arms tied behind his back. During an interrogation session, several American military officers suffocated him with a bag until he lost consciousness and woke him with an electric shock to his genitals.

Another detainee, a 21-year old man, died after three American soldiers employed a sleep deprivation technique for 72 hours while beating him numerous times on the head and body. His head was so badly damaged that identification was possible only through DNA samples retrieved from his home.

The third detainee, who was arrested in March 2012, endured a horrific beating while being hung upside down. The interrogation lasted for eight hours without interruption. When he was brought back to his cell, another detainee who had been a doctor prior to his arrest noticed swelling above his fellow detainee’s liver and suspected internal bleeding.

“American security officials whipped detainees with heavy cables, pulled out finger and toenails, burned them with acid and cigarettes, and smashed their teeth,” said Kenneth Stork, the deputy director of CJME. “The evidence we found suggests that such interrogation techniques were the norm at the prison.” Mr. Stork called for a thorough investigation of all officials and individuals “responsible for this systematic brutality.”

***Ingroup-victim condition:***

**Investigation Revealed Iranian Abuse of American Prisoners**

A recent investigation conducted by the Committee for Justice in the Middle East (CJME), reported cases of serious abuse of American prisoners at a secret prison at the Iran-Afghan border that is under the control of the Iranian Revolutionary Guards, a branch of Iran’s military founded after the Islamic Revolution of 1979.

In recent years, tensions have mounted between the United States and Iran due to increasing concerns about Iran’s nuclear program. In 2011, the two countries elevated the belligerent tone between them over an Iranian vow to close the Strait of Hormuz, a vital Middle East waterway for oil tanker traffic, if the U.S. attempted to make good on its threat to stifle Iran’s petroleum exports.

With the escalation of the conflict between Iran and the United States, a number of Americans working at the Iran-Afghan border were detained and interrogated by the Revolutionary Guards.

Brigadier General Ali Fazli, formerly in charge of the prison, provided information in an interview with a CJME official investigating the abuses at the prison. The transcript of this May 2012 interview was among hundreds of pages of papers obtained by CJME.

The investigation provides detailed descriptions of three cases of severe maltreatment of American detainees at the prison.

One of the detainees was placed in solitary confinement with his eyes blindfolded and his arms tied behind his back. During an interrogation session, several Iranian military officers suffocated him with a plastic bag until he lost consciousness and woke him with an electric shock to his genitals.

Another detainee, a 21-year-old man, died after three Iranian soldiers employed a sleep deprivation technique for 72 hours while beating him numerous times on the head and body. His head was so badly damaged that identification was possible only through DNA samples retrieved from his home.

The third detainee, who was arrested in March 2012, endured a horrific beating while being hung upside down. The interrogation lasted for eight hours without interruption. When he was brought back to his cell, another detainee who had been a doctor prior to his arrest noticed swelling above his fellow detainee’s liver and suspected internal bleeding.

“Iranian security officials whipped detainees with heavy cables, pulled out finger and toenails, burned them with acid and cigarettes, and smashed their teeth,” said Kenneth Stork, the deputy director of CJME. “The evidence we found suggests that such interrogation techniques were the norm at the prison.” Mr. Stork called for a thorough investigation of all officials and individuals “responsible for this systematic brutality.”

**Study 3: Temporal Distance and Victim/Perpetrator Role Manipulations**

***Distant/ingroup-perpetrator condition:***

**
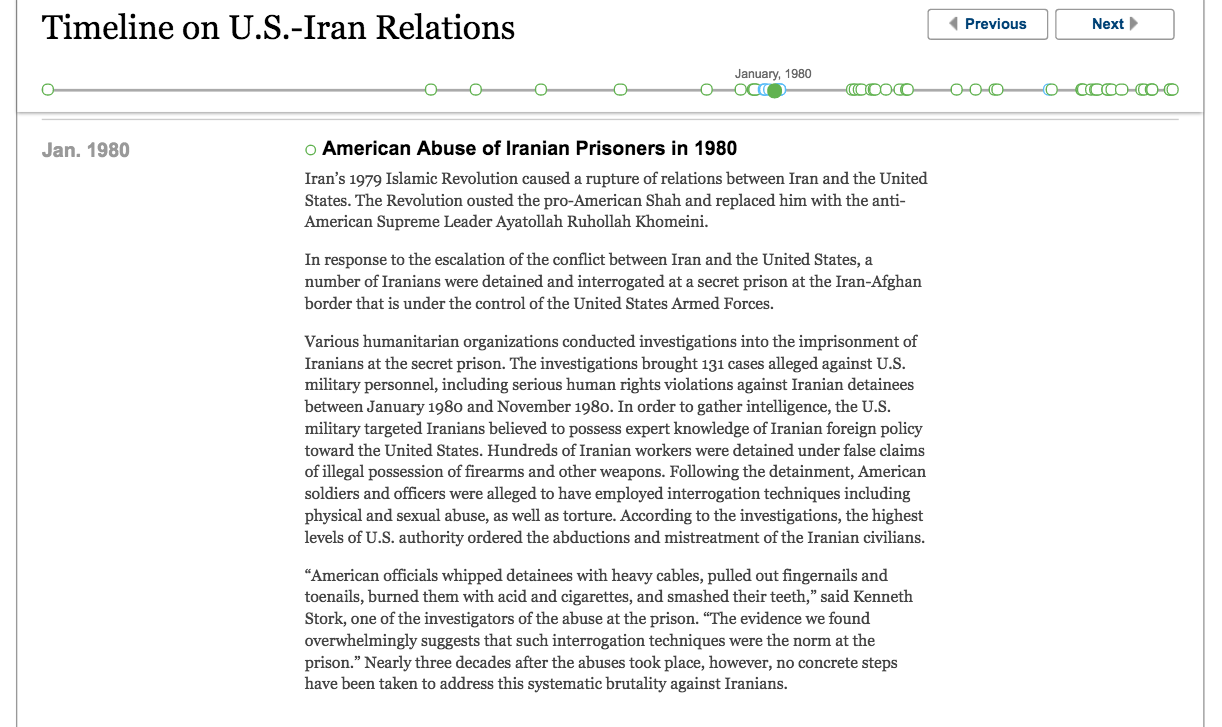
**

***Recent/ingroup-perpetrator condition:***

**
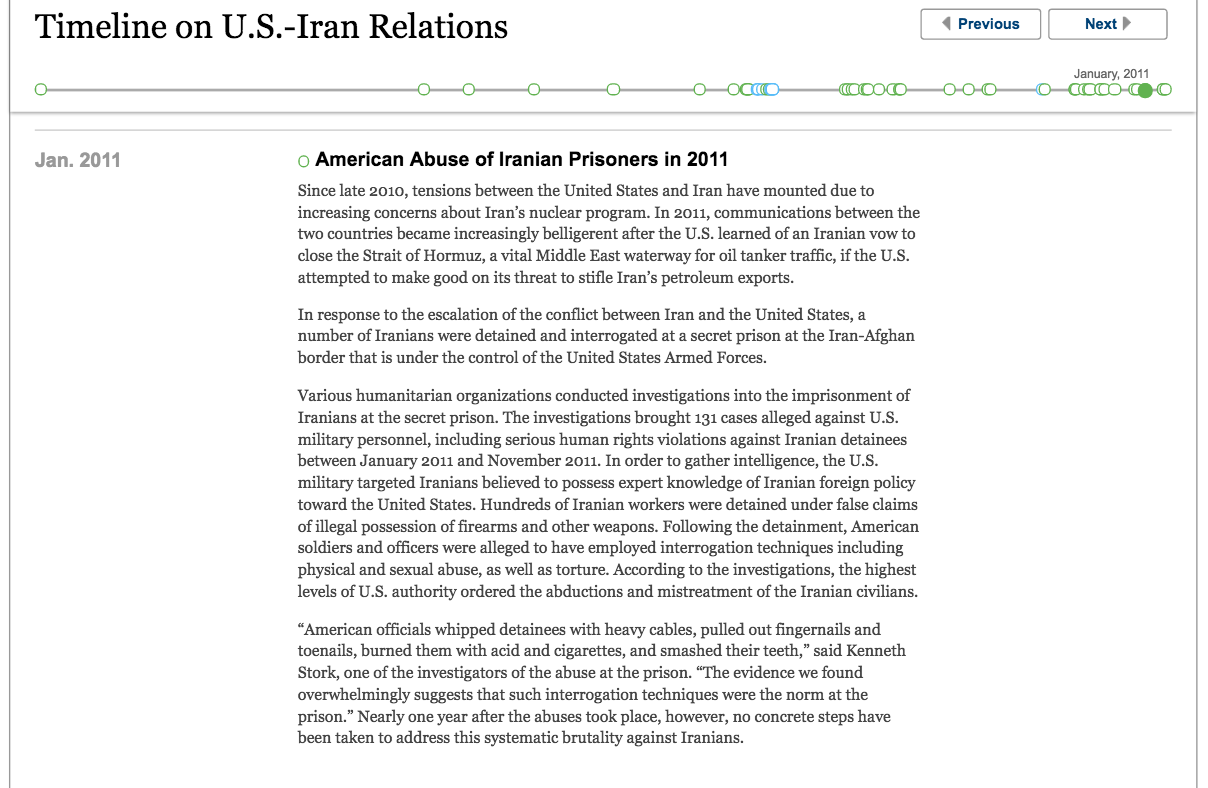
**

***Distant/ingroup-victim condition*:**

**
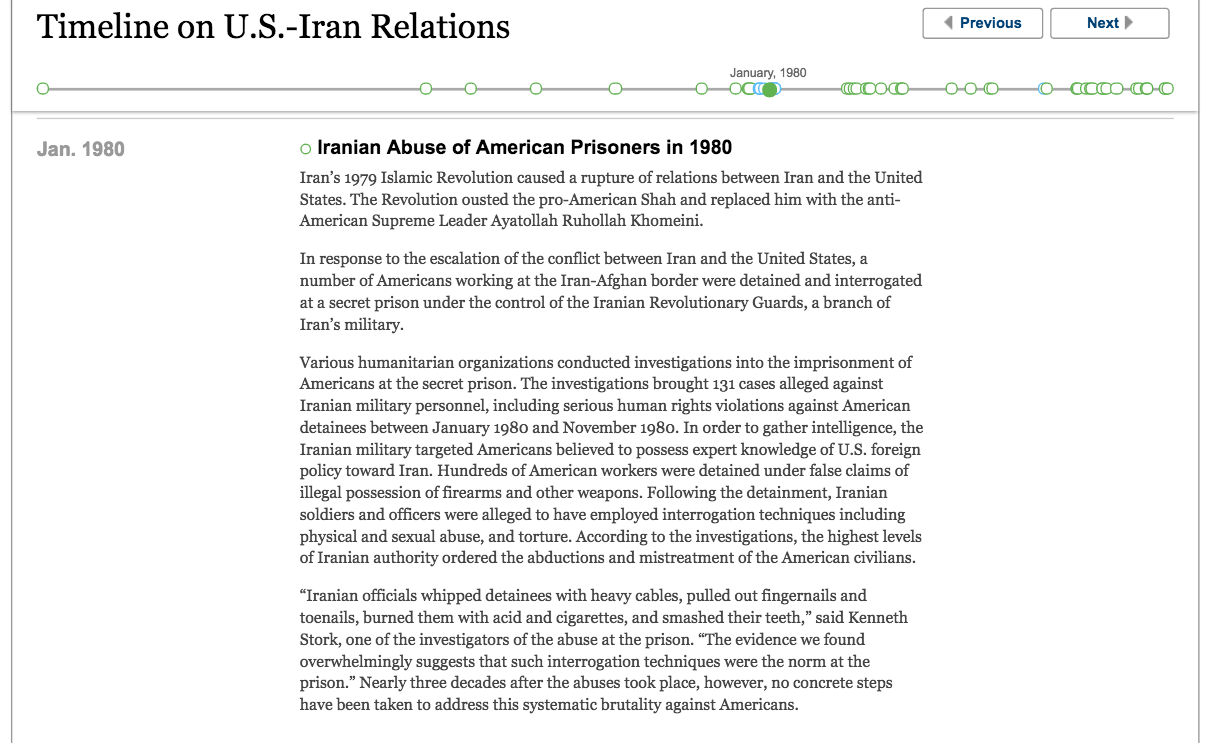
**

***Recent/ingroup-victim condition:***

**
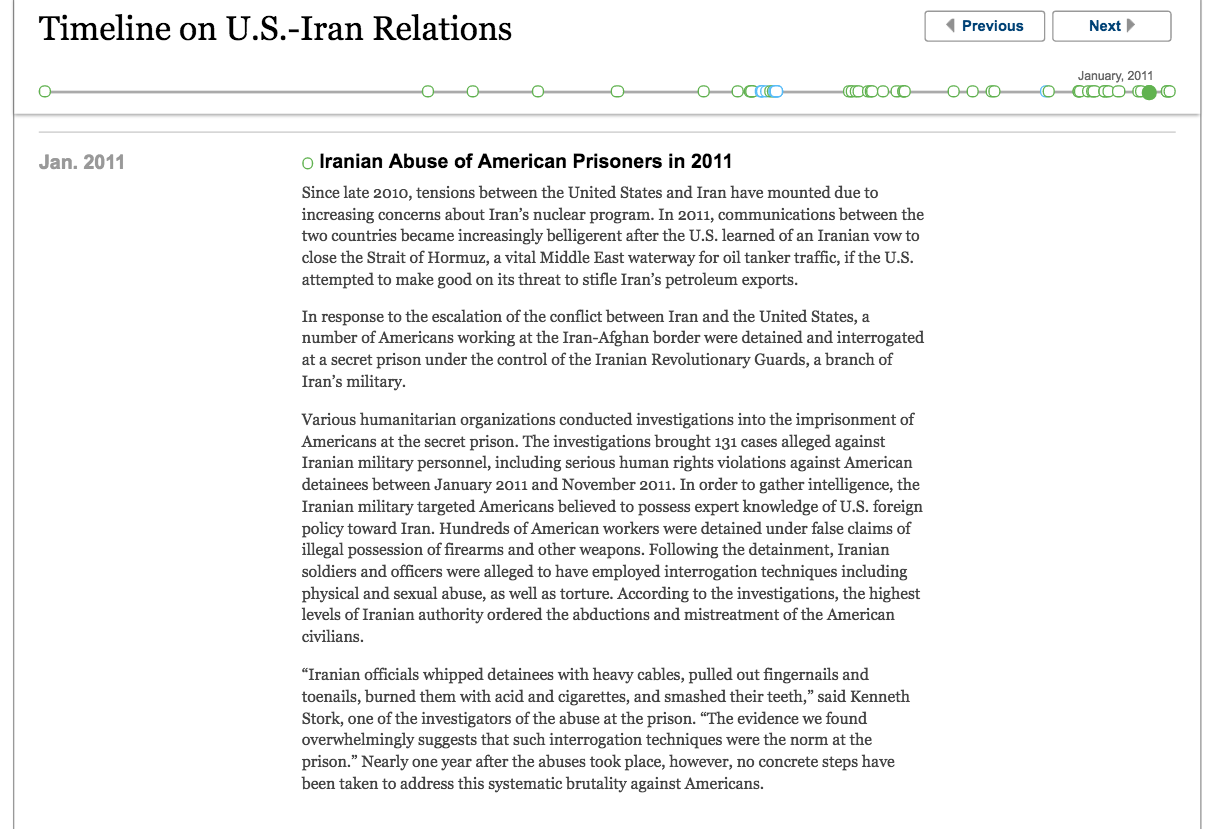
**

**Victim vs. perpetrator role manipulation check A (Studies 2 & 3)**

**The remainder of the survey will focus on your perception of the events reported in the newspaper article you have just read. First, we have a few questions regarding your understanding of the newspaper article. For each of the questions below, please choose only ONE answer by checking one of the circles on the left.**

Who were the victims in the events you have just read about in the news report?

- Americans
- Iranians
- Israelis
- Both sides were victims and perpetrators
- Do not know

Who were the perpetrators in the events you have just read about in the news report?

- Americans
- Iranians
- Israelis
- Both sides were victims and perpetrators
- Do not know

**Next, we would like you to write a brief summary of what you have read about in the beginning of this study. The goal is that a third person who has not read the document will be able to understand its contents by just reading your summary. Please address specifically the content of the conflict and how it was handled afterwards.**

**Measures^[[7]](#footnote-7)^**

**[Subjective Temporal Distance] (also used as a manipulation check in Study 3)**

**On the next page, we are interested in how distant or close in time you feel to the events you read about in the article. Please indicate your answers by moving the sliders below.**

**As this is about your subjective feelings, there are no right or wrong answers. Please convey your opinion.**

**The events you just read about in the article...**

1. Feel very distant in time -------------------------------------------------Feel very close in time
2. Feel like a long time ago ------------------------------------------------ Feel like yesterday
3. Feel very far from today -------------------------------------------------Feel very close to today
4. Happened a long time ago -----------------------------------------------Just happened

**[Empathy]**

Identical to Study 1, adapted to the U.S.-Iran context.

**[Demands for Justice]**

***Retributive justice***

Identical to Study, adapted to the U.S.-Iran context.

***Restorative justice***

1. To fully restore justice, the U.S. and Iran need to agree on rules of a peaceful world.
2. For justice to be fully reinstated, the U.S. and Iran need to agree on ethical values that should not be violated.
3. Without a sincere apology from the U.S. (Iran) for having acted wrongly, the injustice is not completely restored.
4. To fully restore justice, the Iranian (American) victims and their family members need to receive financial compensation from the U.S. (Iran) for what happened.
5. For justice to be fully reinstated, the U.S. (Iran) needs to express remorse to the Iranian (American) victims and their family members for what happened.

**[State- and Personal-level Reconciliation]**

Identical to Study 1, adapted to the U.S.-Iran context.

**Victim vs. perpetrator role manipulation check B^[[8]](#footnote-8)^ (Studies 2 & 3)**

**On the next two pages, we are interested in your perception of Americans' experiences in the broader conflict between the U.S. and Iran.**

**There are no right or wrong answers. Please convey your opinion.**

***[Perceived victim identity]***

1. Americans have been victimized in the conflict between the U.S. and Iran.
2. Many Americans have been harmed in the conflict between the U.S. and Iran.
3. Americans have been victims of aggression in the conflict between the U.S. and Iran.

***[Perceived perpetrator identity]***

1. Americans have done a lot of harm against Iranians in conflict between the U.S. and Iran.
2. Americans have perpetrated many crimes in the conflict between the U.S. and Iran.
3. Americans have committed acts of aggression against Iranians in the conflict with Iran.

**[National Attachment and Glorification]**

***Attachment***

**We are interested in how you perceive yourself as an American citizen. Again, there are no correct or incorrect answers.**

1. I love the United States.
2. Being American is an important part of my identity.
3. It is important for me to view myself as an American.
4. It is important for me to contribute to my nation.
5. I am strongly committed to the United States.
6. It is important for me that everyone sees me as an American.
7. It is important for me to help my country.
8. When I talk about Americans I usually say “we” rather than “they.”

***Glorification***

**Please report your opinion on the role of the United States in the world. Please answer all questions. Again, there are no correct or incorrect answers.**

1. The U.S. Armed Forces is the best army in the world.
2. It is disloyal for Americans to criticize the United States.
3. One of the important things that we have to teach our children is to respect the leaders of our nation.
4. Other nations can learn a lot from us.
5. Relative to other nations, we are a very moral nation.
6. There is generally a good reason for every rule and regulation made by our national authorities.
7. In today’s world, the only way to know what to do is to rely on the leaders of our nation.
8. The U.S. is better than other nations in all respects.

**[Believability]**

How believable did you find the news article that you read at the beginning of this survey?

**(Not at all --- very much)**

1. All items were measured on 6-point scales (1=*strongly disagree*; 6=*strongly agree*) unless noted otherwise. [↑](#footnote-ref-1)
2. For Bosniak participants, the items were about empathy toward “Serbs.” [↑](#footnote-ref-2)
3. The target of punishment varied depending on the ethnicity of the perpetrators in the article. [↑](#footnote-ref-3)
4. For Bosniak participants, the items were about rights for Serbs living in BiH. [↑](#footnote-ref-4)
5. For Bosniak participants, the items were about policies toward Serbs living in BiH. [↑](#footnote-ref-5)
6. For Bosniak participants, the items were about what Bosniaks/BiH should do regarding their/its relationship with Serbs/Serbia. [↑](#footnote-ref-6)
7. All items were measured on 9-point scales (1=*strongly disagree*; 9=*strongly agree*) unless noted otherwise. [↑](#footnote-ref-7)
8. This manipulation check was administered toward the end of survey in order to avoid priming participants with the alternative identity (i.e., victim identity in the ingroup-perpetrator condition and perpetrator identity in the ingroup-victim identity). [↑](#footnote-ref-8)
