# Supplementary material for: Close or Distant Past? The Role of Temporal Distance in Responses to Intergroup Violence From Victim and Perpetrator Perspectives
Source: Pers Soc Psychol Bull. 2020 Aug 1;47(4):657–72. doi: 10.1177/0146167220945890 (PMC7961862; doi:10.1177/0146167220945890)
Supplement: Temporal_distance_Supplementary_document_06-12-2020 – Supplemental material for Close or Distant Past? The Role of Temporal Distance in Responses to Intergroup Violence From Victim and Perpetrator Perspectives [file Temporal_distance_Supplementary_document_06-12-2020.docx]

### **Data Exclusions**

**Study 1**

The data screening procedure resulted in an exclusion of 36 Serb participants because they did not understand or pay sufficient attention to the manipulation material (as indicated by their summaries of this material and incorrect answers to the attention checks), 23 participants because they spent less than 20 seconds reading the manipulation material (less than 10% of the total sample were below this cut-off), and another 16 participants because they spent significantly more time reading the material compared to the rest of the sample (i.e., statistical outliers).

In the Bosnian sample, six participants were excluded because they did not understand or pay sufficient attention to the manipulation material (as indicated by their summaries of this material and incorrect answers to the attention checks) and twelve participants were excluded because they did not identify as Muslims. Because the study was conducted in paper-and-pencil format, we were unable to record the time that participants spent reading the manipulation material, and thus made no exclusion based on reading time.^[[1]](#footnote-1)^

**Study 2**

The data screening procedure resulted in an exclusion of 36 participants who did not understand or pay sufficient attention to the manipulation material (as indicated by their incorrect answers to the attention check questions, wrong summaries of manipulation material, or the little time they spent reading the material), seven who spent significantly more time reading the material compared to the rest of the sample, five who spent less than 10 minutes completing the entire survey (less than half of the average time that participants took), and two who raised suspicion about the credibility of the article in their summaries.

**Study 3** followed the exact same data screening procedure as Study 2.

### **Supplementary Analysis 1**

### **Perceived believability of the ingroup-role manipulation (Study 1)**

Perceived believability was submitted as a DV to a GLM with ethnicity and ingroup role as IVs. The analysis yielded a significant main effect of ingroup role, such that participants found the news report more believable when it portrayed the ingroup as the victim (*M* = 4.28, *SD* = 1.30) rather than the perpetrator (*M* = 3.38, *SD* = 1.43), *F*(1, 436) = 50.80, *p* < .001, *η_p_^2^ =* 0.10 (*LCI* = .06, *UCI* = .15). This main effect was further qualified by an interaction with ethnicity, *F*(1, 436) = 19.29, *p* < .001, *η_p_^2^ =* 0.04 (*LCI* = .02, *UCI* = .08). Simple effects revealed that Bosniak participants found the news report more believable when it portrayed the ingroup as the victim (*M* = 4.54, *SD* = 1.37) rather than the perpetrator (*M* = 3.08, *SD* = 1.57), *t*(436) = 8.12, *p* < .001. By contrast, this difference was only marginally significant among Serbs (*M_victim_ =* 4.01, *M_perpetrator_ =* 3.66), *t*(436) = 1.93, *p* = .053. The main effect of ethnicity was not significant, *F*(1, 436) = .04, *p* = .845, *η_p_^2^ <* 0.001.

**Supplementary Analysis 2**

### **Effects of ethnicity and ingroup-role manipulation (Study 1)**

**Demands for retributive justice.** The same analyses with demands for retributive justice (*α* = .91, *M* = 3.96, *SD* = 1.57) as the DV produced a significant main effect of ethnicity, *F*(1, 439) = 34.05, *p* < .001, *η_p_^2^ =* 0.07 (*LCI* = .04, *UCI* = .11), such that Bosniaks were more supportive of retributive justice (*M* = 4.34, *SD* = 1.66) than Serbs (*M* = 3.59, *SD* = 1.39). There was also a significant main effect of ingroup role on retributive justice, *F*(1, 439) = 177.69, *p* < .001, *η_p_^2^ =* 0.29 (*LCI* = .23, *UCI* = .34). When the ingroup was portrayed as the victim, participants demanded more retributive justice (*M* = 4.75, *SD* = 1.26) compared to when the ingroup was portrayed as the perpetrator (*M* = 3.15, *SD* = 1.44). The two main effects were further qualified by a significant interaction between them, *F*(1, 439) = 37.57, *p* < .001, *η_p_^2^ =* 0.08 (*LCI* = .04, *UCI* = .12). Analyses of simple effects revealed that among Bosniaks, reading about ingroup suffering significantly increased their demands for retributive justice (*M* = 5.45, *SD* = .70) compared to reading about ingroup-committed transgressions (*M* = 3.13, *SD* = 1.55), *t*(439) = 13.68, *p* < .001. The same pattern occurred among Serbs, but to a much lesser degree (*M_victim_ =* 4.02, *M_perpetrator_ =* 3.16), *t*(439) = 5.12, *p* < .001.

**Support for outgroup rights.** We first reverse scored the three items tapping refusal to grant rights to the outgroup, and created a composite score reflecting support for rights for the outgroup members currently living in the participants’ own country (*α* = .82, *M* = 4.51, *SD* = .98). The analyses yielded a significant main effect of ethnicity, *F*(1, 437) = 5.50, *p* = .002, *η_p_^2^ =* 0.01 (*LCI* = .001, *UCI* = .03). Bosniaks were less willing to grant rights to the outgroup members (*M* = 4.40, *SD* = .97) than Serbs (*M* = 4.62, *SD* = .98). The main effect of ingroup role was also significant, *F*(1, 437) = 8.28, *p* = .004, *η_p_^2^ =* 0.02 (*LCI* = .003, *UCI* = .04). When the ingroup was portrayed as the victim, participants were less supportive of outgroup rights (*M* = 4.38, *SD* = 1.02) compared to when the ingroup was portrayed as the perpetrator (*M* = 4.65, *SD* = .92). The interaction between ethnicity and ingroup role did not reach significance, *F*(1, 437) = 1.14, *p* = .287, *η_p_^2^ <* 0.01 (*LCI* < .001, *UCI* = .02).

**Support for policies toward the outgroup.** We first reverse scored the four anti-outgroup policies, and created a composite score reflecting support for pro-outgroup policies (*α* = .82, *M* = 3.78, *SD* = 1.05). The analyses yielded a significant main effect of ethnicity, *F*(1, 437) = 132.54, *p* < .001, *η_p_^2^ =* 0.23 (*LCI* = .18, *UCI* = .29). Bosniaks were less supportive of pro-outgroup policies (*M* = 3.27, *SD* = .87) than Serbs (*M* = 4.28, *SD* = .97). The main effect of ingroup role was also significant, *F*(1, 437) = 16.54, *p* < .001, *η_p_^2^ =* 0.04 (*LCI* = .01, *UCI* = .07). When the ingroup was portrayed as the victim, participants were less supportive of personal-level reconciliation (*M* = 3.59, *SD* = 1.08) compared to when the ingroup was portrayed as the perpetrator (*M* = 3.98, *SD* = 1.00). The interaction between ethnicity and ingroup role did not reach significance, *F*(1, 437) = .15, *p* = .698, *η_p_^2^ <* 0.01 (*LCI* < .001, *UCI* = .01).

**Attitudes toward the ICTY.** The same analyses with attitudes toward the ICTY as the DV (*M* = 2.29, *SD* = .78) yielded a significant main effect of ethnicity, *F*(1, 437) = 129.76, *p* < .001, *η_p_^2^ =* 0.23 (*LCI* = .17, *UCI* = .28). Bosniaks indicated more positive attitudes toward the ICTY (*M* = 2.67, *SD* = .69) than Serbs (*M* = 1.29, *SD* = .69). However, the ingroup-role manipulation did not significantly affect participants’ subjective temporal distance (*M_victim_ =* 2.30, *M_perpetrator_ =* 2.29), *F*(1, 437) = .09, *p* = 0.76, *η_p_^2^* < .01 (*LCI* < .001, *UCI* = .01). The interaction between ethnicity and intergroup role also did not reach significance, *F*(1, 437) = .16, *p* = 0.691, *η_p_^2^* < .01 (*LCI* < .001, *UCI* = .01).

**State-level reconciliation.** We first reverse scored the two items tapping resistance to state-level reconciliation, thus creating a composite score for support for state-level reconciliation at the state level (*α* = .88, *M* = 4.57, *SD* = 1.16). The analyses with support for state-level reconciliation as the DV produced a significant main effect of ethnicity, *F*(1, 437) = 49.11, *p* < .001, *η_p_^2^ =* 0.10 (*LCI* = .06, *UCI* = .15), such that Bosniaks reported less support for reconciliation at the state level (*M* = 4.20, *SD* = 1.24) than Serbs (*M* = 4.93, *SD* = .93). There was also a significant main effect of ingroup role on state-level reconciliation, *F*(1, 437) = 25.67, *p* < .001, *η_p_^2^ =* 0.06 (*LCI* = .03, *UCI* = .09). When the ingroup was portrayed as the victim, participants were less supportive of state-level reconciliation (*M* = 4.30, *SD* = 1.28) compared to when the ingroup was portrayed as the perpetrator (*M* = 4.84, *SD* = .95). The two main effects were further qualified by a significant interaction between ethnicity and ingroup role, *F*(1, 437) = 4.18, *p* = .042, *η_p_^2^ =* 0.01 (*LCI* < .01, *UCI* = .03). Analyses of simple effects revealed that among Bosniaks, reading about ingroup suffering reduced their openness to state-level reconciliation (*M* = 3.85, *SD* = 1.35) compared to reading about ingroup-committed transgressions (*M* = 4.58, *SD* = 1.00), *t*(437) = -5.01, *p* < .001. The same pattern occurred among Serbs, but again to a much lesser degree (*M_victim_ =* 4.77, *M_perpetrator_ =* 5.08), *t*(437) = -2.15, *p* = .033.

**Personal-level reconciliation.** We first reverse scored the two items tapping resistance to personal-level reconciliation, thus creating a composite score for willingness to reconcile with the outgroup at the personal level (*α* = .89, *M* = 4.25, *SD* = 1.31). The analyses with support for personal-level reconciliation as the DV produced a significant main effect of ethnicity, *F*(1, 437) = 59.32, *p* < .001, *η_p_^2^ =* 0.12 (*LCI* = .08, *UCI* = .17), such that Bosniaks were less willing to personally reconcile with the outgroup members (*M* = 3.79, *SD* = 1.33) than Serbs (*M* = 4.69, *SD* = 1.12). The main effect of ingroup role was again significant, *F*(1, 437) = 13.81, *p* < .001, *η_p_^2^ =* 0.03 (*LCI* = .01, *UCI* = .06). When the ingroup was portrayed as the victim, participants were less supportive of personal-level reconciliation (*M* = 4.02, *SD* = 1.41) compared to when the ingroup was portrayed as the perpetrator (*M* = 4.48, *SD* = 1.16). The two main effects were further qualified by a marginally significant interaction, *F*(1, 437) = 2.89, *p* = .090, *η_p_^2^ =* 0.01 (*LCI* < .001, *UCI* = .02). Among Bosniaks, reading about ingroup suffering reduced their openness to personal-level reconciliation (*M* = 3.49, *SD* = 1.37) compared to reading about ingroup-committed transgressions (*M* = 4.12, *SD* = 1.22), *t*(437) = -3.82, *p* < .001. In contrast, Serbs did not differ significantly in their support for personal-level reconciliation depending on the ingroup-role manipulation (*M_victim_ =* 4.58, *M_perpetrator_ =* 4.81), *t*(437) = -1.43, *p* = .153.

**Empathy.** The GLM with empathy toward the outgroup (*α* = .92, *M* = 2.76, *SD* = 1.46) as the DV produced a significant main effect of ethnicity, *F*(1, 435) = 51.40, *p* < .001, *η_p_^2^ =* 0.11 (*LCI* = .06, *UCI* = .15), such that Bosniaks reported lower levels of empathy toward members of the outgroup (*M* = 2.33, *SD* = 1.38) than Serbs (*M* = 3.18, *SD* = 1.42). There was also a significant main effect of ingroup role on empathy, *F*(1, 435) = 175.44, *p* < .001, *η_p_^2^ =* 0.29 (*LCI* = .23, *UCI* = .34). When the ingroup was portrayed as the victim, participants reported less outgroup empathy (*M* = 2.01, *SD* = 1.18) compared to when the ingroup was portrayed as the perpetrator (*M* = 3.52, *SD* = 1.32). The two main effects were further qualified by a significant interaction between ethnicity and ingroup role, *F*(1, 435) = 4.53, *p* = .034, *η_p_^2^ =* 0.01 (*LCI* < .001, *UCI* = .03). Analyses of simple effects revealed that among Bosniaks, reading about ingroup suffering reduced their feelings of empathy toward the outgroup (*M* = 1.49, *SD* = .80) compared to reading about ingroup-committed transgressions (*M* = 3.22, *SD* = 1.30), *t*(435) = -10.81, *p* < .001 (see Table 1 for the means and SDs of all DVs in each condition). The same pattern occurred among Serbs, but to a lesser degree (*M_victim_ =* 2.54, *M_perpetrator_ =* 3.79), *t*(435) = -7.91, *p* < .001.

### **Supplementary Analysis 3**

### **Ingroup-role manipulation checks (Study 2)**

**Perceived victim and perpetrator identity.** Three items measured perceived victimhood of the ingroup in the U.S.-Iran conflict (e.g., “*Americans have been victimized in the conflict between the U.S. and Iran.*”). Three items measured perceived perpetrator identity (e.g., “*Americans have perpetrated many crimes in the conflict between the U.S. and Iran*”). This manipulation check was administered toward the end of the study because responding to the statements about American victimhood might undermine the effectiveness of the ingroup-role manipulation in the ingroup-perpetrator condition, and responding to the statements about American perpetration might undermine the manipulation in the ingroup-victim condition.

**Manipulation check results.** To examine whether the ingroup role manipulation affected participants’ perceived victim or perpetrator identity, *perceived victim identity* (*α* = .91, *M* = 5.92, *SD* = 2.03) and *perceived perpetrator identity* (*α* = .95, *M* = 5.60, *SD* = 2.15) were submitted as DVs to GLMs with ingroup role as the IV. There was a significant effect of ingroup role on perceived victim identity, *F*(1, 224) = 9.93, *p* = .002, *η_p_^2^* = .04 (*LCI* = .01, *UCI* = .09). Participants in the ingroup-victim condition identified more as the victims of the conflict (*M* = 6.34, *SD* = 1.78) than participants in the ingroup-perpetrator condition (*M* = 5.51, *SD* = 2.18). Perceived perpetrator identity was also significantly affected by the ingroup-role manipulation, *F*(1, 224) = 22.01, *p* < .001, *η_p_^2^* = .09 (*LCI* = .04, *UCI* = .15). Participants in the ingroup-perpetrator condition identified more as the perpetrators of the conflict (*M* = 6.24, *SD* = 1.97) than participants in the ingroup-victim condition (*M* = 4.95, *SD* = 2.14). Overall, the results of the manipulation checks indicated that the ingroup-role manipulation successfully increased American participants’ perceived victim or perpetrator status of the U.S. in the conflict with Iran.

A mixed factorial ANOVA with the perceived victim vs. perpetrator identity measure as a within-subject IV and ingroup role as a between-subject IV revealed a significant interaction between identity measure and ingroup role, *F*(1, 224) = 30.11, *p* < .001. This interaction suggests that participants in the ingroup-victim condition identified more as victims rather than perpetrators (*M_victimID_ =* 6.34, *M_perpetratorID_ =* 4.95), whereas participants in the ingroup-perpetrator condition identified more as perpetrators rather than victims (*M_victimID_ =* 5.51, *M_perpetratorID_ =* 6.24).

**Supplementary Analysis 4**

**Effects of condition on glorification and attachment (Study 2)**

Neither attachment, *F*(1, 224) = 0.05, *p* = .824, *η_p_^2^* < .01, nor glorification, *F*(1, 224) = 2.98, *p* = .086, *η_p_^2^* = .01, was significantly affected by condition, thus allowing us to use them, together with condition, as continuous IVs in the subsequent GLMs. To this end, attachment and glorification were centered (Aiken & West, 1991; Cohen, Cohen, West, & Aiken, 2003).

**Supplementary Analysis 5**

**Effects of ingroup role and glorification (Study 2)**

Table 2 displays least-squares means and SEs of all DVs in each condition by glorification.

**Retributive Justice.** The same analysis with demands for retributive justice as the DV (*α* = .93, *M* = 6.15, *SD* = 1.90) yielded a significant interaction between ingroup role and glorification, *F*(1, 221) = 64.37, *p* < .001, *η_p_^2^* = .23 (*LCI* = .15, *UCI* = .30). Simple effects revealed that high glorifiers were *more* supportive of retributive justice when their ingroup was the victim (*M* = 7.01) than when it was the perpetrator (*M* = 5.04), *t*(221) = -6.24, *p* < .001. In contrast, low glorifiers exhibited the opposite pattern, demanding *less* retributive justice when the ingroup was the victim (*M* = 5.27) than when it was the perpetrator (*M* = 6.89), *t*(221) = 5.06, *p* < .001. No other effects reached significance, *Fs*(1, 221) < 1.00, *ps* > .320, *η_p_^2^s* < .01 (*LCIs* < .001, *UCIs* < .03).

**Restorative Justice.** The same analysis with demands for restorative justice as the DV (*α* = .82, *M* = 6.85, *SD* = 1.50) again yielded a significant interaction between ingroup role and glorification, *F*(1, 221) = 38.46, *p* < .001, *η_p_^2^* = .14 (*LCI* = .08, *UCI* = .22). High glorifiers were *more* supportive of restorative justice when the ingroup was the victim (*M* = 7.06) rather than the perpetrator (*M* = 6.12), *t*(221) = -3.65, *p* < .001. In contrast, low glorifiers exhibited the opposite pattern, demanding *less* restorative justice when the ingroup was the victim (*M* = 6.32) rather than the perpetrator (*M* = 7.65), *t*(221) = 5.07, *p* < .001. The main effect of attachment was significant, indicating that attachment was positively associated with restorative justice, *β* = .22, *F*(1, 221) = 6.31, *p* = .013, *η_p_^2^* = .03. No other effects reached significance, *Fs*(1, 221) < 2.30, *ps* > .130, *η_p_^2^s* < .01 (*LCIs* < .001, *UCIs* < .05).

**Willingness to Reconcile.** The analysis with willingness to reconcile (*α* = .92, *M* = 5.24, *SD* = 1.78) as the DV yielded a significant main effect of ingroup role, *F*(1, 221) = 6.92, *p* = .009, *η_p_^2^* = .03 (*LCI* < .01, *UCI* = .08). When the ingroup was the victim, participants were less willing to reconcile with the outgroup (*M* = 4.87, *SD* = 1.82) compared to when the ingroup was the perpetrator (*M* = 5.61, *SD* = 1.67). The main effect of ingroup role was further qualified by a significant interaction with glorification, *F*(1, 221) = 3.89, *p* = .050, *η_p_^2^* = .02 (*LCI* < .01, *UCI* = .05). High glorifiers were less willing to reconcile when the ingroup was the victim (*M* = 3.91) than when it was the perpetrator (*M* = 4.83), *t*(221) = 3.28, *p* = .001. In contrast, low glorifiers did not differ significantly depending on condition (*M_victim_ =* 6.11, *M_perpetrator_ =* 6.25), *t*(221) = .48, *p* = .633. The main effect of glorification was also significant, indicating that glorification was negatively associated with willingness to reconcile, *β* = -.51, *F*(1, 221) = 39.17, *p* < .001, *η_p_^2^* = .15. The main effect of attachment was not significant, *F*(1, 221) = .12, *p* = .730, *η_p_^2^* < .01 (*LCI* < .001, *UCI* = .02).

**Empathy.** The same analysis with empathy toward the outgroup as the DV (*α* = .94, *M* = 4.45, *SD* = 2.22) yielded a significant main effect of ingroup role, *F*(1, 221) = 66.14, *p* < .001, *η_p_^2^* = .23 (*LCI* = .15, *UCI* = .30). When the ingroup was the victim, participants were less empathic toward the outgroup (*M* = 3.39, *SD* = 1.90) compared to when the ingroup was the perpetrator (*M* = 5.49, *SD* = 1.92). As expected, the main effect of ingroup role was further qualified by a significant interaction with glorification, *F*(1, 221) = 4.61, *p* = .033, *η_p_^2^* = .02 (*LCI* < .01, *UCI* = .06). Simple effects revealed that high glorifiers were less empathetic toward the outgroup when their ingroup was the victim (*M* = 2.36) than when it was the perpetrator (*M* = 4.72), *t*(221) = 7.34, *p* < .001. Low glorifiers exhibited the same pattern, but to a much lesser degree (*M_victim_ =* 4.73, *M_perpetrator_ =* 6.11), *t*(221) = 4.24, *p* < .001. The main effect of glorification was significant, indicating that glorification was negatively associated with empathy, *β* = -.42, *F*(1, 221) = 32.63, *p* < .001, *η_p_^2^* = .13. The main effect of attachment was not significant, *F*(1, 221) = .01, *p* = .918, *η_p_^2^* < .01 (*LCI* < .001, *UCI* < .01)**.**

**Supplementary Analysis 6**

### **Ingroup-role manipulation check (Study 3)**

Across conditions, participants’ perceived victim and perpetrator identity were negatively correlated, *r*(443) = -.34, *p* < .001. *Perceived victim identity* (*α* = .91, *M* = 6.08, *SD* = 1.83) was significantly affected by the role manipulation, *F*(1, 440) = 28.56, *p* < .001, *η_p_^2^* = .06 (*LCI* = .03, *UCI* = .10). Participants in the ingroup-victim condition identified more as the victims of the conflict (*M* = 6.50, *SD* = 1.51) than participants in the ingroup-perpetrator condition (*M* = 5.67, *SD* = 2.03). This main effect was further qualified by an interaction with the distance manipulation, *F*(1, 440) = 11.15, *p* = .001, *η_p_^2^* = .02 (*LCI* = .01, *UCI* = .05). When the ingroup was the victim, participants in the distant condition reported significantly less perceived victimhood (*M* = 6.25) than participants in the close condition (*M* = 6.81), *t*(440) = 2.34, *p* = .020. When the ingroup was the perpetrator, in contrast, participants exhibited the opposite pattern (*M_distant_* = 5.91, *M_close_* = 5.34), *t*(440) = -2.38, *p* = .018. The main effect of the distance manipulation was not significant, *F*(1, 440) < .01, *p* = .976, *η_p_^2^* < .01 (*LCI* < .001, *UCI* < .01).

Participants’ *perceived perpetrator identity* (*α* = .94, *M* = 5.66, *SD* = 1.87) was also significantly affected by the ingroup-role manipulation, *F*(1, 439) = 23.99, *p* < .001, *η_p_^2^* = .05 (*LCI* = .02, *UCI* = .09). Participants in the ingroup-perpetrator condition identified more as the perpetrators of the conflict (*M* = 6.07, *SD* = 1.78) than participants in the ingroup-victim condition (*M* = 5.26, *SD* = 1.86). No other effects reached significance, *Fs*(1, 439) < 2.26, *ps* > .130, *η_p_^2^s* < .01 (*LCIs* < .001, *UCIs* < .03).

A mixed factorial ANOVA with the perceived victim vs. perpetrator identity measure as a within-subject IV, and the ingroup role and temporal distance manipulations as between-subject IVs revealed a significant interaction between identity measure and ingroup role, *F*(1, 439) = 40.74, *p* < .001. This interaction suggests that participants in the ingroup-victim condition identified more as victims rather than perpetrators (*M_victimID_ =* 6.50, *M_perpetratorID_ =* 5.26), whereas participants in the ingroup-perpetrator condition identified more as perpetrators rather than victims (*M_victimID_ =* 5.67, *M_perpetratorID_ =* 6.07). The three-way interaction between identity measure, ingroup role, and the distance manipulation was also significant, *F*(1, 439) = 9.10, *p* = .003. This interaction indicated that the significant effect of distance on perceived victimhood (reported above) was significantly stronger in magnitude than the nonsignificant effect of distance on perceived perpetrator identity.

**Supplementary Analysis 7**

**Temporal distance manipulation check**

We first examined whether the ingroup role and the temporal distance manipulations affected participants’ perceived temporal distance. The analysis with subjective temporal distance (*α* = .96, *M* = 5.54, *SD* = 2.14) as the DV revealed a significant main effect of the temporal distance manipulation, *F*(1, 440) = 103.12, *p* < .001, *η_p_^2^* = .19 (*LCI* = .14, *UCI* = .24). Participants in the distant conditions reported feeling temporally further away from the transgression (*M* = 6.36, *SD* = 1.89) than participants in the close conditions (*M* = 4.48, *SD* = 1.98). No other effects reached significance, *Fs*(1, 440) < .98, *ps* > .325, *η_p_^2^s* < .01 (*LCI* < .001, *UCI* < .02).

We also tested whether subjective temporal distance was affected by an interaction between the role manipulation and glorification. The main effect of ingroup role was further qualified by an interaction with glorification, *F*(1, 435) = 7.26, *p* = .007, *η_p_^2^* = .02 (*LCI* < .01, *UCI* = .04). Analyses of simple effects revealed that high glorifiers perceived the prisoner abuses as temporally closer when their ingroup was the victim (*M* = 5.35) than the perpetrator (*M* = 5.96), *t*(435) = 2.34, *p* = .020 (see Tables 3a and 3b for LS means and SEs of all DVs by condition and glorification). Low glorifiers, in contrast, did not differ significantly on subjective temporal distance depending on their ingroup’s role (*M_victim_ =* 5.42, *M_perpetrator_ =* 5.02), *t*(435) = -1.51, *p* = .132. The main effect of glorification was marginally significant, *F*(1, 435) = 2.75, *p* = .098, *η_p_^2^* < .01, indicating that glorification was somewhat positively associated with subjective temporal distance, *β* = .10. No other effects reached significance, *Fs*(1, 435) < .80, *ps* > .380, *η_p_^2^s* < .01.

### **Supplementary Analysis 8**

**Effects of manipulations on attachment and glorification (Study 3)**

Neither attachment (*α* = .95, *M* = 6.84, *SD* = 1.77), nor glorification (*α* = .90, *M* = 4.79, *SD* = 1.72), was affected by the ingroup role or temporal distance manipulations, *Fs*(1, 440) < 2.42, *ps* > .120, *η_p_^2^s* < .01 (*LCIs* < .001, *UCIs* < .03); nor were they affected by the interaction between ingroup role or temporal distance, *Fs*(1, 440) < .20, *ps* > .660, *η_p_^2^s* < .01 (*LCIs* < .001, *UCIs* < .01). Thus, in the subsequent GLMs, the ingroup role (victim vs. perpetrator) and temporal distance (distant vs. close) manipulations were entered as categorical IVs, glorification as a continuous moderator, and attachment as a covariate.

**Effects of ingroup role, temporal distance, and glorification (additional results; Study 3)**

Tables 3a-b display least-squares means and SEs of all DVs in each condition by ingroup role and temporal distance at high and low levels of glorification.

**Retributive justice.** The main effect of the role manipulation was significant, *F*(1, 435) = 28.13, *p* < .001, *η_p_^2^* = .06 (*LCI* = .03, *UCI* = .10). Participants demanded more retributive justice when the ingroup was the victim (*M* = 6.31, *SD* = 1.68) than when it was the perpetrator (*M* = 5.49, *SD* = 2.02). Replicating the findings of Study 2, the main effect of ingroup role was further qualified by an interaction with glorification, *F*(1, 435) = 78.81, *p* < .001, *η_p_^2^* = .15 (*LCI* = .10, *UCI* = .20). High glorifiers demanded more retributive justice when the ingroup was the victim (*M* = 6.81) than when it was the perpetrator (*M* = 4.58), *t*(435) = -10.03, *p* < .001. Low glorifiers, in contrast, exhibited the opposite pattern, demanding less retributive justice when the ingroup was the victim (*M* = 5.86) than when it was the perpetrator (*M* = 6.44), *t*(435) = 2.64, *p* = .009. The main effect of the temporal distance manipulation was also significant, *F*(1, 435) = 42.80, *p* < .001, *η_p_^2^* = .09 (*LCI* = .05, *UCI* = .13), such that participants demanded less retributive justice in the distant condition (*M* = 5.45, *SD* = 1.99) than in the close condition (*M* = 6.49, *SD* = 1.60). There was again a main effect of glorification, *F*(1, 435) = 4.19, *p* = .041, *η_p_^2^* = .01, indicating that glorification was negatively associated with demand for retributive justice (*β* = -.12). No other main or interaction effects reached significance, *Fs*(1, 435) < .90, *ps* > .350, *η_p_^2^s* < .01 (*LCIs* < .001, *UCIs* < .02).

**Restorative justice.** The analysis also revealed a main effect of ingroup role, *F*(1, 435) = 16.93, *p* < .001, *η_p_^2^* = .04 (*LCI* = .01, *UCI* = .07). Participants demanded more restorative justice when the ingroup was the victim (*M* = 6.98, *SD* = 1.27) than when it was the perpetrator (*M* = 6.45, *SD* = 1.74). Again replicating the findings in Study 2, the main effect of ingroup role was again qualified by an interaction with glorification, *F*(1, 435) = 27.02, *p* < .001, *η_p_^2^* = .06 (*LCI* = .03, *UCI* = .10). High glorifiers demanded more restorative justice when the ingroup was the victim (*M* = 6.88) than when it was the perpetrator (*M* = 5.61), *t*(435) = -6.58, *p* < .001. Low glorifiers, in contrast, did not differ significantly depending on ingroup role (*M_victim_ =* 7.11, *M_perpetrator_ =* 7.28), *t*(435) = .84, *p* = .402. The main effect of the temporal distance manipulation was also significant, *F*(1, 435) = 10.99, *p* = .001, *η_p_^2^* = .02 (*LCI* = .01, *UCI* = .05), such that participants demanded less restorative justice in the distant condition (*M* = 6.51, *SD* = 1.72) than in the close condition (*M* = 6.98, *SD* = 1.23). The main effects of glorification and attachment were also significant, *Fs*(1, 435) > 6.30, *ps* < .012, *η_p_^2^s* > .01, indicating that glorification was negatively (*β* = -.30) and attachment positively (*β* = .20) associated with demands for restorative justice. No other effects reached significance, *Fs*(1, 435) < 1.02, *ps* > .316, *η_p_^2^s* < .01 (*LCIs* < .001, *UCIs* < .02).

**Willingness to reconcile.** There was a marginally significant main effect of the temporal distance manipulation on reconciliation, *F*(1, 435) = 3.09, *p* = .079, *η_p_^2^* = .01 (*LCI* < .01, *UCI* = .03). Participants were somewhat more willing to reconcile in the distant condition (*M* = 5.37, *SD* = 1.77) than in the close condition (*M* = 5.18, *SD* = 1.71). There was a marginally significant interaction between the distance manipulation and glorification, *F*(1, 435) = 3.50, *p* = .062, *η_p_^2^* = .01 (*LCI* < .01, *UCI* = .03). Whereas low glorifiers reported more reconciliatory attitudes in the distant conditions (*M* = 6.71) than in the close conditions (*M* = 6.21), *t*(435) = 2.58, *p* = .010, high glorifiers did not significantly differ depending on when the violence occurred (*M_distant_ =* 4.08, *M_close_ =* 4.10), *t*(435) = -.10, *p* = .917. The main effects of glorification and attachment were also significant, *Fs*(1, 435) > 10.90, *ps* < .002, *η_p_^2^s* > .02, indicating that glorification was negatively (*β* = -.68) and attachment positively (*β* = .18) associated with willingness to reconcile. No other effects reached significance, *Fs*(1, 435) < 1.25, *ps* > .268, *η_p_^2^s* < .01 (*LCIs* < .001, *UCIs* < .02).

**Empathy.** The analysis also revealed a significant main effect of the role manipulation, *F*(1, 435) = 132.83, *p* < .001, *η_p_^2^* = .23 (*LCI* = .18, *UCI* = .28). Participants were less empathic toward the outgroup when the ingroup was the victim (*M* = 3.25, *SD* = 1.90) than when it was the perpetrator (*M* = 5.20, *SD* = 2.10). The main effect of ingroup role was further qualified by a significant interaction with the distance manipulation, *F*(1, 435) = 23.05, *p* < .001, *η_p_^2^* = .05 (*LCI* = .02, *UCI* = .09). Analyses of simple effects indicated that when the ingroup was the victim, participants in the distant condition expressed more empathy (*M* = 3.75) than participants in the close condition (*M* = 2.79), *t*(435) = -4.10, *p* < .001. When the ingroup was the perpetrator, in contrast, participants in the distant condition expressed less empathy (*M* = 4.85) than participants in the close condition (*M* = 5.48), *t*(435) = 2.70, *p* = .007. There was also a significant interaction between the distance manipulation and glorification, *F*(1, 435) = 5.28, *p* = .022, *η_p_^2^* = .01 (*LCI* < .01, *UCI* = .03). Whereas low glorifiers reported more empathy in the distant conditions (*M* = 5.57) than in the close conditions (*M* = 5.03), *t*(435) = 2.33, *p* = .020, high glorifiers did not significantly differ on empathy depending on when the transgression occurred (*M_distant_ =* 3.03, *M_close_ =* 6.50), *t*(435) = -.95, *p* = .340. The main effect of glorification also reached significance, *F*(1, 435) = 84.83, *p* < .001, *η_p_^2^* = .07, indicating that glorification was negatively associated with outgroup empathy (*β* = -.49). The main effect of attachment was marginally significant, *F*(1, 435) = 3.11, *p =* .079, *η_p_^2^* = .01, indicating that attachment was positively associated with empathy (*β* = .10). No other main or interaction effects reached significance, *Fs*(1, 435) < 1.20, *ps* > .260, *η_p_^2^s* < .01 (*LCIs* < .001, *UCIs* < .02).

### **Supplementary Analysis 9**

### **Alternative model: Empathy as a mediator**

**Study 1.** We also tested the path model where subjective temporal distance was entered as the exogenous variable, empathy toward the outgroup as the mediator, and demand for retributive justice, support for pro-outgroup policies, attitudes toward the ICTY, and willingness to reconcile as outcome variables. Because the four outcome variables were closely related, their error terms were allowed to freely co-vary. The overall model with all parameters freely estimated in the four groups provided an excellent fit to the data, *χ^2^*(16) = 13.08, *p* = .667, *CFI* = 1.00, *SRMSR* = .04, *RMSEA* < .01, *GFI* = .99, *NFI* = .98. The model was also an adequate fit for the data from each subgroup.

Among Bosniaks in the ingroup-victim condition (*SRMSR* = .05, *GFI* = .99, *NFI* = .97; Figure 1a), temporal distancing from the war positively predicted empathy toward the outgroup. Empathy, in turn, predicted demands for retributive justice negatively, support for pro-outgroup policy positively, and willingness to reconcile positively. Empathy was not significantly associated with attitudes toward the ICTY. Among Serbs in the ingroup-perpetrator condition (*SRMSR* = .03, *GFI* = .99, *NFI* = .99; Figure 1b), in contrast, temporal distancing from the war negatively predicted empathy toward the outgroup. Empathy, in turn, positively predicted demands for retributive justice, support for pro-outgroup policy, favorable attitudes toward the ICTY, and willingness to reconcile. Importantly, the two subgroups differed significantly on the relation between subjective temporal distance and empathy, *t* = 3.03, *p* *=* .002. Although the model also fit the data very well in the other two subgroups (Bosniak/perpetrator: *SRMSR* = .05, *GFI* = .98, *NFI* = .97; Serb/victim: *SRMSR* = .01, *GFI* = 1.00, *NFI* = 1.00), subjective temporal distance did not significantly predict outgroup empathy, *β*s < .14, *ps* > .130.

We further tested the indirect effects of temporal distance on attitudes toward justice and reconciliation via empathy. The analyses revealed significant indirect effects of temporal distance on demands for retributive justice, support for pro-outgroup policy, and reconciliation among Bosniaks in the ingroup-victim condition, .19 > *β*s > .10, *ps* < .025, but not in the ingroup-perpetrator condition, *β*s < .09, *ps* > .140. Among Serbs, the indirect effects of temporal distance on all four outcome variables were marginally significant in the ingroup-perpetrator condition, -.12 < *β*s < -.05, *ps* < .085, but not in the ingroup-victim condition, *β*s > -.04, *ps* > .480. Furthermore, the indirect effects were (marginally) different between Bosniaks in the victim condition and Serbs in the perpetrator condition, *ts* > 1.90, *ps* *<* .052, supporting the hypothesis that temporal distance plays different roles in predicting intergroup outcomes between victim and perpetrator group members.

**Study 2.** In the alternative path model, glorification was entered as the exogenous variable, subjective temporal distance as the “step 1” mediator, empathy toward the outgroup as the “step 2” mediator, and demand for (retributive and restorative) justice and willingness to reconcile as the outcome variables, controlling for attachment as another exogenous variable. To be consistent with the GLMs, the path model also included direct paths from glorification to empathy, justice demands, and willingness to reconcile. Because retributive and restorative justice are two closely related constructs, their error terms were allowed to freely co-vary, and so were the error terms of the two justice measures and reconciliation. The statistical models for the ingroup victim and perpetrator conditions are depicted with standardized path coefficients in Figures 2a and 2b.

The overall model with all parameters freely estimated in the two groups provided an excellent fit to the data, *χ^2^*(17) = 16.36, *p* = .499, *CFI* = 1.00, *SRMSR* = .04, *RMSEA* < .01, *GFI* = .98, *NFI* = .98. The model was also an adequate fit for the data in each subgroup (ingroup-victim: *SRMSR* = .05, *GFI* = .97, *NFI* = .97; ingroup-perpetrator: *SRMSR* = .03, *GFI* = .98, *NFI* = .99). However, the fit comparison between the two subgroups also revealed that the ingroup-victim condition contributed more to the overall chi-square (74%) than the ingroup-perpetrator condition (26%), suggesting that the model fit for the perpetrator condition was better than that for the victim condition.

In the ingroup-victim condition (Figure 2a), glorification negatively predicted temporal distancing. However, temporal distance did not significantly predict outgroup empathy. Empathy negatively predicted demands for retributive and restorative justice, and positively predicted willingness to reconcile. In addition, glorification predicted empathy negatively, both types of justice positively, and reconciliation negatively. In the ingroup-perpetrator condition (Figure 2b), in contrast, glorification positively predicted more temporal distancing, which predicted reduced empathy toward the outgroup. Empathy, in turn, positively predicted demands for both retributive and restorative justice, as well as willingness to reconcile. In addition, glorification also negatively predicted empathy, retributive justice, and reconciliation, but not restorative justice.

The multi-group path analysis further tested the indirect effects of glorification on justice demands and willingness to reconcile via subjective temporal distance and empathy as sequential mediators. The analyses revealed significant indirect effects of glorification on all three outcome variables in the ingroup-perpetrator condition (retributive justice: *β* = -.05, *p* *=* .044; restorative justice: *β* = -.06, *p* *=* .042; reconciliation: *β* = -.05, *p* *=* .044), but not in the ingroup-victim condition, *ps* > .500. Although the indirect effects in the ingroup-victim condition were not significantly different from zero, they were (marginally) different from those in the ingroup-perpetrator condition, *t*s < -1.70, *ps* < .08, thus supporting the main hypothesis that temporal distance (in conjunction with glorification) plays different roles in predicting attitudes toward justice and reconciliation via empathy among victim and perpetrator group members.

**Study 3.** To test the moderated mediation model in which temporal distance affects attitudes toward justice and reconciliation differently depending on ingroup role and glorification via empathy (Figure 3), we conducted a series of mediation analyses using PROCESS with 5,000 bootstrap samples and 95% confidence intervals. Temporal distance was dummy coded with the close condition as the reference group. In each analysis, the dummy coded distance manipulation was introduced as the IV (predictor), empathy as the mediator, ingroup role and glorification as moderators, and each of the three final outcomes as the DV (Hayes, 2013, model 12).^[[2]](#footnote-2)^

***Demands for Retributive Justice.*** The indirect effects of the distance manipulation on retributive justice through empathy were significant at *low* levels of glorification in the ingroup-victim condition (*b* = .23, *LCI* = .063, *UCI* = .489), and in the opposite direction at *high* levels of glorification in the ingroup-perpetrator condition (*b* = -.10, *LCI* = -.285, *UCI* = -.002). These significant indirect effects indicate that when the intergroup violence was distant (rather than close) in time, low glorifiers in the victim condition were more empathic toward outgroup perpetrators (*b* = 1.63, *p* < .001), which in turn predicted lowered demands for retributive justice (*b* = -.30, *p* < .001). High glorifiers in the perpetrator condition, by contrast, were less empathic toward the outgroup victims (*b* = -.66, *p* = .005), which in turn predicted less support for retributive justice (*b* = .52, *p* < .001).

In addition to the hypothesized indirect effects, there was also a significant indirect effect of temporal distance on retributive justice via empathy at low levels of glorification in the ingroup-perpetrator condition (*b* = -.08, *LCI* = -.225, *UCI* = -.005). As expected, the indirect effect at high levels of glorification in the ingroup-victim condition was not significant (*b* = .04, *LCI* = -.022, *UCI* = .149). Importantly, the overall index of *moderated* moderated mediation (see Hayes, 2017, for a comparison between three types of moderated mediation: *partial* moderated mediation, *conditional* moderated mediation, and *moderated* moderated mediation) was significant, *b* = -.09, *LCI* = -.244, *UCI* = -.003, indicating that the moderation of the indirect effect of temporal distance on retributive justice by ingroup role depended on glorification.

***Demands for Restorative Justice*.** The indirect effects of the distance manipulation on restorative justice through empathy were significant at *low* levels of glorification in the ingroup-victim condition (*b* = .46, *LCI* = .249, *UCI* = .740), and in the opposite direction at *high* levels of glorification in the ingroup-perpetrator condition (*b* = -.20, *LCI* = -.459, *UCI* = -.010). As expected, the indirect effect at low levels of glorification in the ingroup-perpetrator condition was not significant (*b* = -.15, *LCI* = -.345, *UCI* = .005), nor was it significant at high levels of glorification in the ingroup-victim condition (*b* = -.08, *LCI* = -.055, *UCI* = .229). The overall moderated mediation index was again significant, *b* = -.17, *LCI* = -.376, *UCI* = -.006.

***Willingness to Reconcile.*** The indirect effects of the distance manipulation on reconciliation through empathy were again significant at *low* levels of glorification in the ingroup-victim condition (*b* = .82, *LCI* = .458, *UCI* = 1.180), and marginally significant at 94% CI in the opposite direction at *high* levels of glorification in the ingroup-perpetrator condition (*b* = -.36, *LCI* = -.732, *UCI* = -.008). In addition to the hypothesized indirect effects, the analysis also revealed a marginally significant (94% CI) indirect effect at low levels of glorification in the ingroup-perpetrator condition (*b* = -.27, *LCI* = -.558, *UCI* = -.003). As expected, the indirect effect was not significant at high levels of glorification in the ingroup-victim condition (*b* = .14, *LCI* = -.118, *UCI* = .375). The overall moderated mediation index was marginally significant at 94% CI, *b* = -.30, *LCI* = -.619, *UCI* = -.007.

**Tables**

|  | Bosniaks | | | | | |  | |  | Serbs | | | | | | | | |
| --- | --- | --- | --- | --- | --- | --- | --- | --- | --- | --- | --- | --- | --- | --- | --- | --- | --- | --- |
|  | Victim | |  | Perpetrator | |  | |  | Victim | | |  | | | Perpetrator | |  | |
|  | *M* | *SD* | | *M* | *SD* | | | *ES*(*r*) | *M* | | *SD* | | *M* | | | *SD* | | *ES*(*r*) |
| Temporal distance | 39.54_a_ | 29.15 | | 40.84_a_ | 27.88 | | | 0.02 | 56.32_a_ | | 26.48 | | | 50.07_a_ | | 27.74 | | 0.08 |
| Retributive justice | 5.45_a_ | 0.70 | | 3.13_b_ | 1.55 | | | 0.55 | 4.02_a_ | | 1.31 | | | 3.16_b_ | | 1.33 | | 0.23 |
| Outgroup rights | 4.22_a_ | 1.02 | | 4.59_b_ | 0.88 | | | 0.13 | 4.54_a_ | | 1.00 | | | 4.71_a_ | | 0.95 | | 0.06 |
| Pro-outgroup policies | 3.09_a_ | 0.90 | | 3.48_b_ | 0.97 | | | 0.15 | 4.12_a_ | | 1.00 | | | 4.44_a_ | | 0.93 | | 0.12 |
| Attitudes toward ICTY | 2.65_a_ | 0.72 | | 2.70_a_ | 0.65 | | | 0.02 | 1.93_a_ | | 0.74 | | | 1.92_a_ | | 0.64 | | 0.003 |
| State-level reconcile | 3.85_a_ | 1.35 | | 4.58_b_ | 1.00 | | | 0.23 | 4.77_a_ | | 1.01 | | | 5.08_b_ | | 0.83 | | 0.10 |
| Personal-level reconcile | 3.49_a_ | 1.37 | | 4.12_b_ | 1.22 | | | 0.18 | 4.58_a_ | | 1.23 | | | 4.81_a_ | | 0.83 | | 0.07 |
| Empathy | 1.49_a_ | 0.80 | | 3.22_b_ | 1.30 | | | 0.46 | 2.54_a_ | | 1.26 | | | 3.79_b_ | | 1.29 | | 0.35 |

*Note.* Difference subscripts indicate significant differences within each ethnic group.

Table 1. Means and SDs of all dependent variables by participants’ ethnicity and the ingroup-role manipulation, and effect sizes for differences between the ingroup-victim and the ingroup-perpetrator conditions (Study 1).

|  | High Glorifiers (+1 SD) | | | | | |  | | | Low Glorifiers (-1 SD) | | | | | | | | |  |
| --- | --- | --- | --- | --- | --- | --- | --- | --- | --- | --- | --- | --- | --- | --- | --- | --- | --- | --- | --- |
|  | Victim | |  | Perpetrator | |  | | |  | | Victim | |  | | Perpetrator | | | |  |
|  | *M* | *SE* | | *M* | *SE* | | | *ES*(*r*) | | *M* | | *SE* | | *M* | | *SE* | | | *ES*(*r*) |
| Temporal distance | 4.12_a_ | 0.27 | | 5.05_b_ | 0.30 | | | 0.17 | | 4.95_a_ | | 0.31 | | | 4.17_b_ | | | 0.27 | 0.14 |
| Retributive justice | 7.01_a_ | 0.24 | | 5.04_b_ | 0.27 | | | 0.39 | | 5.27_a_ | | 0.27 | | | 6.89_b_ | | 0.24 | | 0.32 |
| Restorative justice | 7.06_a_ | 0.19 | | 6.12_b_ | 0.22 | | | 0.24 | | 6.32_a_ | | 0.22 | | | 7.65_b_ | | 0.19 | | 0.32 |
| Reconciliation | 3.91_a_ | 0.21 | | 4.83_b_ | 0.24 | | | 0.22 | | 6.11_a_ | | 0.24 | | | 6.25_a_ | | 0.21 | | 0.03 |
| Empathy | 2.36_a_ | 0.24 | | 4.72_b_ | 0.27 | | | 0.44 | | 4.73_a_ | | 0.27 | | | 6.11_b_ | | 0.24 | | 0.27 |

*Note.* Difference subscripts indicate significant differences within high and low glorifiers.

Table 2. Least-squares (LS) means and SEs of all key dependent variables by level of glorification (with attachment controlled for) and condition, and effect sizes (r) for differences between the ingroup-victim and the ingroup-perpetrator conditions (Study 2).

|  | Victim (high glorifiers) | | | | | |  | | Perpetrator (high glorifiers) | | | | | | |  |
| --- | --- | --- | --- | --- | --- | --- | --- | --- | --- | --- | --- | --- | --- | --- | --- | --- |
|  | Close | |  | Distant | |  | |  | | Close | |  | | Distant | |  |
|  | *M* | *SE* | | *M* | *SE* | | | *ES*(*r*) | | *M* | *SE* | | *M* | | *SE* | *ES*(*r*) |
| Retributive justice | 7.21_a1_ | 0.24 | | 6.41_b1_ | 0.22 | | | 0.13 | | 5.34_a2_ | 0.27 | | | 3.81_b2_ | 0.22 | 0.22 |
| Restorative justice | 6.89_a1_ | 0.21 | | 6.87_a1_ | 0.19 | | | 0.004 | | 6.10_a2_ | 0.23 | | | 5.11_b2_ | 0.19 | 0.16 |
| Reconciliation | 3.82_a1_ | 0.20 | | 4.32_b_^†^_1_ | 0.19 | | | 0.09 | | 4.38_a2_^†^ | 0.23 | | | 3.84_b_^†^_2_^†^ | 0.19 | 0.09 |
| Empathy | 2.11_a1_ | 0.25 | | 2.37_a1_ | 0.23 | | | 0.04 | | 4.40_a2_ | 0.29 | | | 3.68_b2_ | 0.23 | 0.10 |

*Note.* Difference letter subscripts indicate significant differences within the ingroup-victim and the ingroup-perpetrator conditions. Difference number subscripts indicate significance differences within the close and the distant conditions. Effect sizes displayed are for simple effects within the ingroup-victim and perpetrator conditions.

^†^ indicates marginal significance.

Table 3a. Least-squares (LS) means and SEs of all key dependent variables by the ingroup role and the temporal distance manipulations at *high* levels of glorification (with attachment controlled for), and effect sizes (r) for differences between the close and distant conditions (Study 3).

|  | Victim (low glorifiers) | | | | | |  | | Perpetrator (low glorifiers) | | | | | | |  |
| --- | --- | --- | --- | --- | --- | --- | --- | --- | --- | --- | --- | --- | --- | --- | --- | --- |
|  | Close | |  | Distant | |  | |  | | Close | |  | | Distant | |  |
|  | *M* | *SE* | | *M* | *SE* | | | *ES*(*r*) | | *M* | *SE* | | *M* | | *SE* | *ES*(*r*) |
| Retributive justice | 6.46_a1_ | 0.25 | | 5.26_b1_ | 0.24 | | | 0.18 | | 6.71_a1_ | 0.24 | | | 6.17_b_^†^_2_ | 0.21 | 0.09 |
| Restorative justice | 7.41_a1_ | 0.22 | | 6.81_b1_ | 0.21 | | | 0.10 | | 7.36_a1_ | 0.21 | | | 7.19_a1_ | 0.18 | 0.03 |
| Reconciliation | 5.85_a1_ | 0.22 | | 6.80_b1_ | 0.21 | | | 0.16 | | 6.57_a2_ | 0.21 | | | 6.61_a1_ | 0.18 | 0.01 |
| Empathy | 3.48_a1_ | 0.27 | | 5.12_b1_ | 0.25 | | | 0.22 | | 6.57_a2_ | 0.25 | | | 6.03_b_^†^_2_ | 0.22 | 0.08 |

*Note.* Difference letter subscripts indicate significant differences within the ingroup-victim and the ingroup-perpetrator conditions. Difference number subscripts indicate significance differences within the close and the distant conditions. Effect sizes displayed are for simple effects within the ingroup-victim and perpetrator conditions.

^†^ indicates marginal significance.

Table 3b. Least-squares (LS) means and SEs of all key dependent variables by the ingroup role and the temporal distance manipulations at *low* levels of glorification (with attachment controlled for), and effect sizes (r) for differences between the close and distant conditions (Study 3).


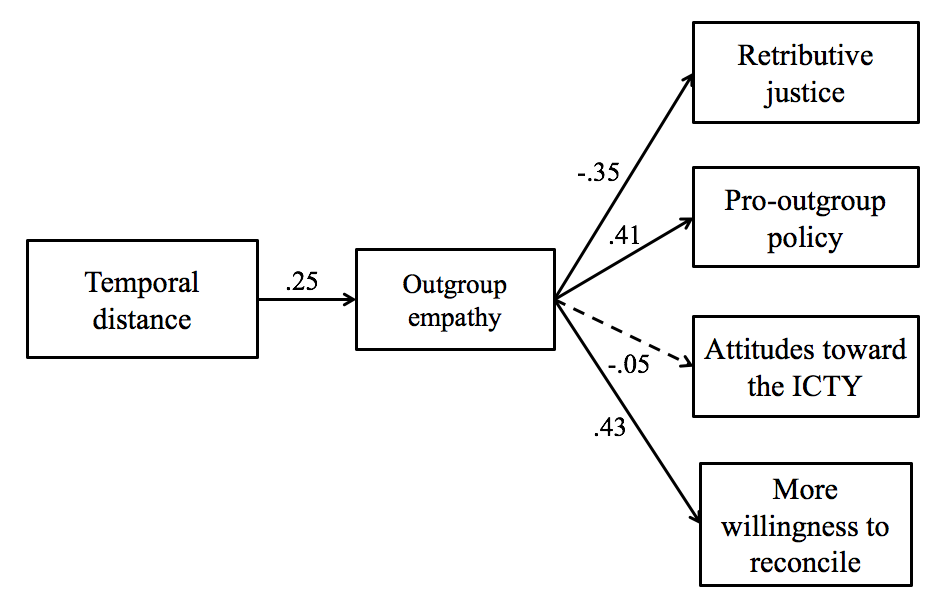


Figure 1a.


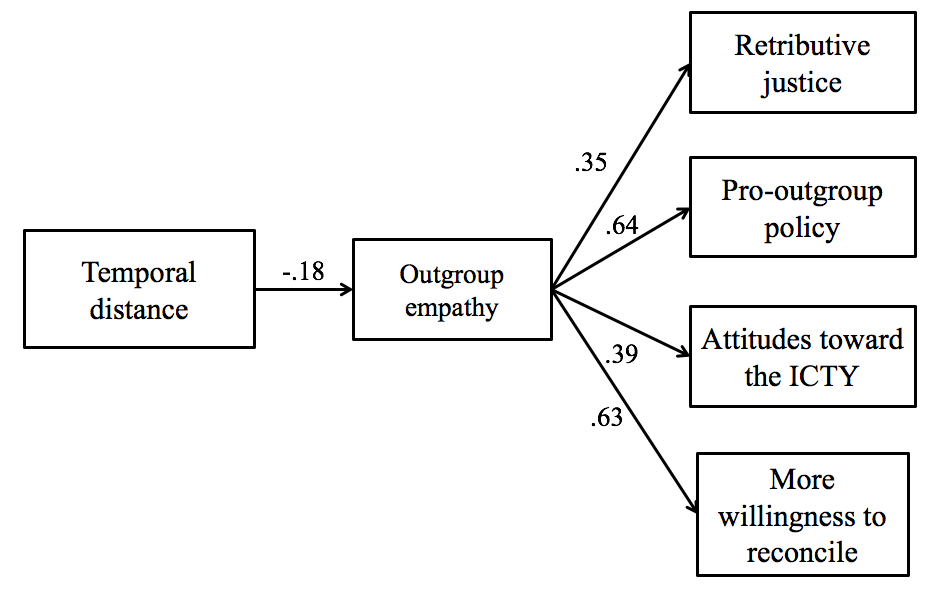


Figure 1b.

Figures 1a and 1b. Statistical models depicting the effects of subjective temporal distance on attitudes toward justice and reconciliation through outgroup empathy among Bosniaks in the ingroup-victim condition (1a) and among Serbs in the ingroup-perpetrator condition (1b). Solid paths were significant; dashed paths were not (Study 1).


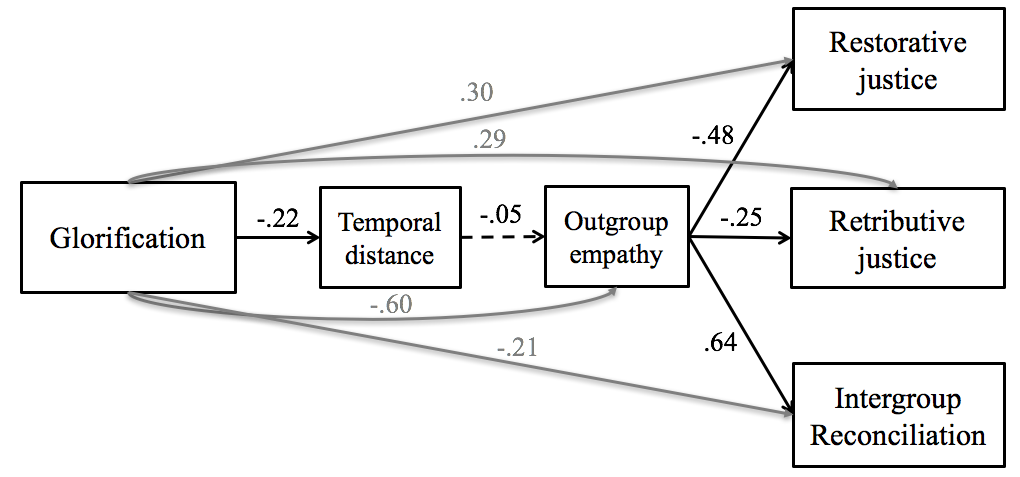


Figure 2a.


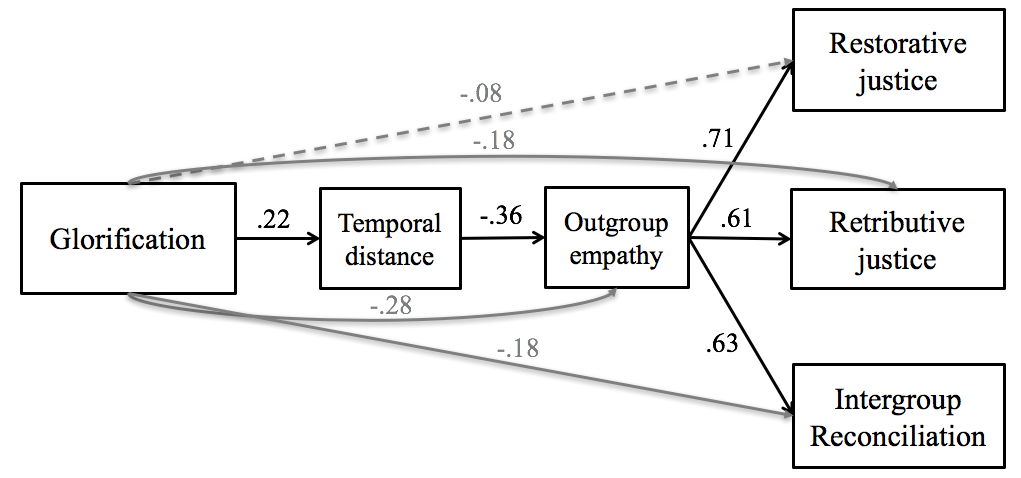


Figure 2b.

Figures 2a and 2b. Statistical models depicting the effects of ingroup glorification on demands for retributive and restorative justice and reconciliation through temporal distance and empathy in the *ingroup-victim condition* (2a) and the *ingroup-perpetrator condition* (2b). Paths displayed in black were central to the hypotheses; paths displayed in gray were not. Solid paths were significant; dashed paths were not (Study 2).


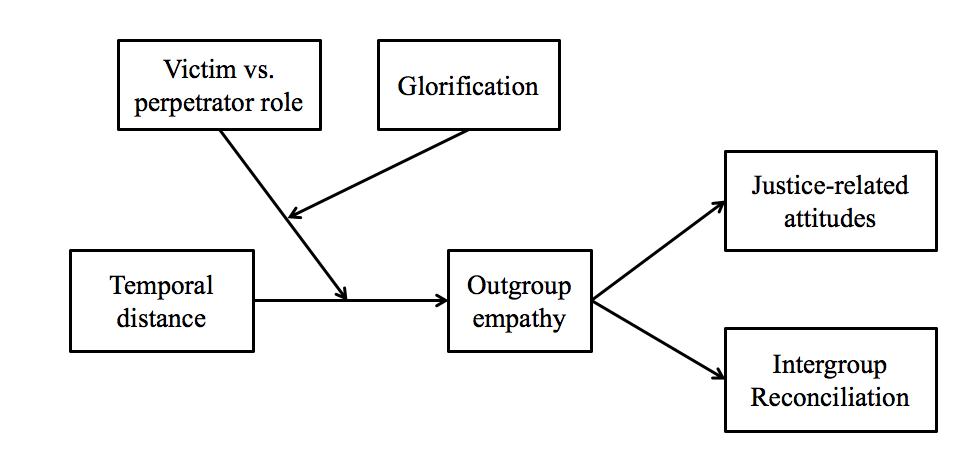


Figure 3. Conceptual model depicting the effects of the temporal distance manipulation on justice-related attitudes and reconciliation through empathy, moderated by the ingroup role manipulation and glorification (Study 3)

1. To be consistent with the Bosniak sample, we also conducted the subsequent analyses without excluding Serb participants based on their reading time. Results remained unchanged. [↑](#footnote-ref-1)
2. Although it would be ideal to test the full model with all three DVs included simultaneously, it was not statistically possible to conduct a multi-group path analysis comparing high and low levels of glorification in combination with ingroup role (e.g., victim/high glorification, victim/low glorification, etc.). Therefore, we carried out moderated mediation analyses using PROCESS Model 12. [↑](#footnote-ref-2)
